# Imbalances in circulating monocyte and high-density lipoprotein cholesterol exacerbates the residual risk of incident myocardial infarction beyond LDL-C: a real-life, prospective cohort study

**DOI:** 10.1186/s12967-025-07028-7

**Published:** 2025-12-30

**Authors:** Dan Wu, Yulong Lan, Xiong Ding, Lois Balmer, Xingang Li, Wei Wang, Shouling Wu, Youren Chen

**Affiliations:** 1 Department of Cardiology, Second Affiliated Hospital of Shantou University Medical College, 69 Dongxia North Road, Jinping District, Shantou, 515041 China; 2 Centre for Precision Health, School of Medical and Health Sciences, Edith Cowan University, Room 521, Building 21/270 Joondalup Drive, Perth, WA 6027 Australia; 3 School of Public Health, Wuhan University, Wuhan, 430072 China; 4 Clinical Research Centre, First Affiliated Hospital of Shantou University Medical College, Shantou, 515041 China; 5 Beijing Key Laboratory of Clinical Epidemiology, School of Public Health, Capital Medical University, Beijing, 100069 China; 6 School of Public Health, Shandong First Medical University & Shandong Academy of Medical Sciences, Tai’an, 271016 China; 7 Department of Cardiology, Kailuan General Hospital, 57 Xinhua East Road, Lubei District, Tangshan, 063000 China

**Keywords:** Residual cardiovascular risk, Monocytes, High-density lipoprotein, Monocyte-to-high-density lipoprotein ratio, Low-density lipoprotein, Inflammation

## Abstract

**Background:**

A sustained oversupply of monocytes and markedly decreased levels of high-density lipoprotein cholesterol (HDL-C) are notable in high-risk groups with residual cardiovascular risk, despite aggressive low-density lipoprotein cholesterol (LDL-C) lowering. This study aimed to explore whether an imbalance in circulating monocyte and HDL-C levels accounts for the residual risk of incident myocardial infarction (MI) within optimal LDL-C levels.

**Methods:**

A total of 48,522 participants free of cardiovascular diseases from a real-life, prospective cohort (Kailuan study) were included. The monocyte count, HDL-C level and monocyte-to-HDL-C ratio (MHR), calculated as time-averaged cumulative and baseline values, were used as the exposures. Time-to-event survival analyses were conducted to examine the association between exposure and MI incidence, especially tests for risk heterogeneity across different LDL-C levels and other potential confounders.

**Results:**

During a median follow-up of 10.06 years, 573 MI events occurred. An elevated time-averaged MHR was positively correlated with clustering of traditional risk factors but inversely correlated with LDL-C levels. After adjusting for potential confounders, each 1-SD increase in the time-averaged monocyte count, HDL-C level and MHR was significantly associated with a risk of 1.18 (95% CI: 1.08–1.28), 0.86 (95% CI: 0.79–0.93) and 1.27 (95% CI: 1.16–1.38), respectively. Individuals with LDL-C levels between 1.8 and 2.6 mmol/L had the lowest MI incidence but the highest MI risk (1.50, 95% CI: 1.27–1.79) with exposure to time-averaged MHR. In contrast, individuals with LDL-C levels ≥ 3.4 mmol/L had the highest MI incidence, but nonsignificant MI risk (HR: 0.99, 95% CI 0.82–1.19) was associated with time-averaged MHR.

**Conclusion:**

Chronic imbalances in monocyte and HDL-C levels are significantly associated with incident MI among individuals with low LDL-C levels. Longitudinally tracing MHR changes and targeting elevated MHR may help to optimize risk assessment and management of MI.

**Supplementary Information:**

The online version contains supplementary material available at 10.1186/s12967-025-07028-7.

## Introduction

Myocardial infarction (MI) is the leading cause of death in developed countries; approximately 550,000 first episodes and 200,000 recurrent episodes of MI occur annually [1, 2]. Notwithstanding the effectiveness of lowering low-density lipoprotein cholesterol (LDL-C) for primary prevention, there is significant residual cardiovascular risk (RCR), especially in high-risk populations, such as those with diabetes, renal dysfunction or chronic autoimmune disease [3–7]. Discovering biomarkers to optimize risk assessment and management is an unmet need for precision medicine [8].

Notably, during these morbidities, a systemic oversupply of circulating monocytes concurrent with significantly decreased levels of high-density lipoprotein cholesterol (HDL-C) has long been observed [7, 9–15]. For example, diabetic dyslipidemia is characterized by typically low levels of HDL-C [12, 13], and hyperglycemia drives monocytosis by activating hematopoietic activity in the bone marrow [14, 15]. Interestingly, both monocyte and HDL-C levels appear to be intrinsically related during atherogenesis. In addition to the inverse cross-sectional correlation between blood monocyte counts and HDL-C levels [7], compelling evidence from experiment-based studies has also demonstrated a biological interplay between them [16, 17]. Raising HDL-C levels in rheumatoid arthritis (RA) [11] or diabetes [18, 19] patients can significantly inhibit monocytosis and reduce residual cardiovascular risk after LDL-C lowering treatment. In this context, it is possible that an imbalance in monocyte and HDL-C levels may account for residual cardiovascular risks and enable precise risk assessment and stratification.

Clearly, disordered hematopoiesis enhances atherothrombosis [20]. Chronically increased monocytosis has been demonstrated to drive the low-grade inflammatory milieu and promote all stages of atherogenesis, including cardiac ischemia [21] and ventricular remodeling postinfarction [20–22]. Notably, the occurrence of MI events also accelerates atherosclerosis and begets MI via mechanisms involving a sustained boost in monocyte production following MI [23]. Thus, monocyte-related biomarkers, e.g., the monocyte-HDL-C ratio [24–27], total monocyte count [28] and monocyte subset [29], have been established as valuable predictors of major adverse cardiovascular events and mortality among patients with existing cardiac ischemic diseases. To further fill in the knowledge gap regarding the association of chronic imbalances in monocyte and HDL-C levels with incident MI among the general population, especially when interacting with other traditional risk factors (LDL-C or diabetes, renal dysfunction, inflammation, etc.), we therefore conducted an analysis based on data from a real-world, prospective cohort in China.

## Methods

### Study participants

The present analysis was based on data from the Kailuan Study (registration number: ChiCTR-TNC-11001489). As previously described [30, 31], this is an ongoing, real-life, community-based and prospectively designed cohort study approved by the Kailuan General Hospital Ethics Committee, China (2006–05). To investigate the risk factors for cardiometabolic diseases, employees and retirees of the Kailuan group have been recruited to participate in a biennial health examination circle since 2006. During each health survey, all participants completed a self-administered questionnaire, underwent a physical examination and provided blood samples for biochemical measurements. For the present analysis, a total of 45,822 participants who participated in three consecutive health surveys between 2006/2007 and 2010/211, had complete data on monocyte counts and lipid profiles and were free of cardiovascular disease (CVD) and cancer were included. A flowchart of the participants is shown in Supplementary Figure 1, and the study design is presented in supplementary eFigure 2.

### Exposure

Monocyte counts were obtained from the routine blood panel and tested using a complete blood count analyzer (Sysmex XT–1800i, Sysmex). Biological tests of lipid profiles were conducted by an automatic analyzer (Hitachi 747; Hitachi, Tokyo, Japan). To assess chronic imbalances in circulating monocyte counts and HDL-C levels, we calculated the time-averaged cumulative monocyte count (CumMON), HDL-C (CumHDL) and the monocyte-to-HDL-C ratio (CumMHR, with MHR calculated as monocyte count/HDL-C) in accordance with a well-established algorithm [32]. For each variable, the time‑averaged cumulative value was calculated as the weighted mean of the average value for each interval, divided by the total exposure period:

$$\begin{aligned}[\text{time }1\!-\!2&*(\text{Value }1 + \text{Value }2)/2 + \text{time }2\!-\!3 \\ &*(\text{Value }2 + \text{Value }3)/2]/\text{time }1\!-\!3 \end{aligned}$$

where Value 1, Value 2 and Value 3 are the values of the studied variables obtained from three consecutive health examinations, while time 1–2, time 2–3 and time 1–3 represent the time intervals between examinations.

### Outcomes

MI [International Classification of Disease, 10th Revision (ICD-10): I21] was defined in accordance with the World Health Organization Multinational Monitoring of Trends and Determinants in Cardiovascular Disease criteria. Ascertainment of MI has been previously delineated [30, 31]. Briefly, potential MI events were ascertained from the Municipal Social Insurance Institution, which covered all the study participants, the discharge registers of all 11 Kailuan hospitals, and a questionnaire survey (biennially since 2006). For suspected MI events identified by the ICD-10 and questionnaire, a panel of 3 experienced physicians reviewed the medical records and adjudicated the cases annually. Survival time for each participant was calculated as the duration from the response date of the baseline survey through the time of incident first MI, death, or the end of follow-up (December 31, 2020). The baseline characteristics of included and excluded participants were represented at Supplementary eTable 1.

### Covariates

We included age, sex, smoking status, alcohol consumption status, physical activity, education level, body mass index (BMI), systolic blood pressure (SBP), total cholesterol (TC), high-sensitivity C-reactive protein (hsCRP), leukocyte count, renal function and prevalent diseases, as well as medication use, as potential confounders. The selection of covariates was based on established CVD risk factors and supported by prior literature [33, 34]. Most covariates included showed meaningful baseline imbalances between case and non-case groups (indicated by standardized mean differences (SMDs) > 0.10 [35, 36]); Supplementary eTable 2). Details of the measurement of the covariables are specified in the Supplementary Methods.

### Statistical analysis

The data on the study covariates were more than 98% complete. The missingness proportion and pattern plots (Supplementary eFigures 3 and 4) indicated a general (non-monotone) missing pattern. Multiple Imputation by Chained Equations (MICE) with 10 imputations was performed using the MI procedure in SAS. Plausible physiological ranges were pre-specified to avoid implausible values. Estimates were combined across datasets using Rubin’s rules (MIANALYZE procedure in SAS) [37, 38]. Continuous normally distributed variables are reported as the mean and standard deviation (SD). Variables with skewed distributions are reported as medians with interquartile ranges (IQRs). Categorical variables are reported as frequencies and percentages (%). Log-transformed values of time-averaged cumulative and baseline monocyte counts, MHR, HDL-C, hsCRP and leucocyte counts were used when they were included as continuous variables in the statistical models. Baseline differences between MI cases and noncases were compared using the chi-square test, an unpaired Student’s *t* test or the Mann‒Whitney U test. Intergroup comparisons across CumMHR quartiles were conducted by one-way analyses of variance, the Kruskal‒Wallis test and the chi-square test, as appropriate.

The absolute risk of incident MI was assessed by calculating both the cumulative incidence and events per 1,0000 person-years. After confirming that the proportionality assumption was met by assessing interactions with time [33], Cox proportional hazards regression was used to estimate adjusted hazard ratios (aHRs) with 95% CIs for incident MI both per SD increment and across increasing quartiles of MHR, monocytes and HDL-C in both time-averaged and baseline patterns. Covariables included in the multivariable adjusted Cox models included sex (male/female), age (continuous), smoking habit (yes or no), alcohol consumption (yes or no), physical activity (low, moderate and high), educational attainment (less than high school or high school and above), BMI (continuous), family history of CVD (yes or no), TC (continuous), SBP (continuous), prevalent diabetes (yes or no) and log-transformed (hsCRP) (continuous) as well as medication use of antihypertensives (yes or no), lipid-lowering drugs (yes or no) and antidiabetics (yes or no). As monocytes are a subset of circulating leukocytes, the model was additionally adjusted for leucocyte counts. Additionally, because the MHR per se is a ratio that indeed reflects an interaction term in a statistical model, we further adjusted for both separate components in an additional model to test the interaction effect. To test whether the CumMHR may account for residual MI risk beyond LDL-C, we specifically investigated CumMHR-associated MI risk across different LDL-C subgroups (LDL-C < 1.8, 1.8 to 2.6, 2.6 to 3.4, ≥ 3.4 mmol/L) [39–41]. In additional subgroup analyses, we examined the risk heterogeneities stratified by prevalent diabetes, prevalent hypertension, prevalent dyslipidemia, prevalent renal dysfunction or subclinical inflammation. Evidence for heterogeneity of risk factors across CumMHR quartiles was tested by means of a likelihood ratio test by adding multiplicative interaction terms to a proportional hazards model. Subgroup and interaction analyses were considered exploratory and not adjusted for multiple comparisons.

Several sensitivity analyses were performed to assess the robustness of the study results. First, to minimize potential confounding by cardiovascular events, we excluded MI onset within the first year of follow-up to address potential reverse causality. Second, to account for the competitive risk of death, the Fine‒Gray model was used to analyze the difference in the risk of MI after controlling for the competing risk of death. Third, we repeated the main analysis based on the raw data without imputation. Fourth, covariates with SMD > 0.1 were identified as meaningfully imbalanced and included in an additional multivariable-adjusted model.

A 2-tailed *P* value < 0.05 was considered to indicate statistical significance, with no adjustment for multiplicity. All the statistical analyses were conducted using SAS (version 9.4; SAS Institute, Cary, North Carolina).

## Results

The baseline characteristics of the study participants are shown across cases and noncases as well as across CumMHR quartiles (Table 1). The study population had a mean age of 52.8 ± 11.9 years, and 37,692 (77.7%) were males. Compared to individuals without MI onset, participants with incident MI were older and mainly male and had higher values of time-averaged and baseline MHR, BMI, blood pressure, TG, TC, LDL-C and hsCRP. In addition, they were more likely to be current smokers and have prevailing diabetes, hypertension or dyslipidemia. A graded increase in the CumMHR was positively correlated with baseline BMI, blood pressure, TG and hsCRP levels but inversely correlated with baseline LDL-C levels.

**Table 1:** Baseline characteristics of the study participants

	Total (*n* = 48,522)	Non-MI (47,949)	MI (*n* = 573)	CumMHR Q1 (*n* = 12,130)	CumMHR Q2 (*n* = 12,131)	CumMHR Q3 (*n* = 12,130)	CumMHR Q4 (*n* = 12,131)
CumMHR, median (IQR)	0.23 (0.17–0.31)	0.23 (0.17–0.31)	0.26 (0.20–0.34)	0.14 (0.12–0.15)	0.20 (0.18–0.21)	0.26 (0.25–0.28)	0.38 (0.34–0.45)
CumMON, median (IQR), 10^9^/L	0.33 (0.26–0.42)	0.33 (0.26–0.42)	0.36 (0.30–0.46)	0.22 (0.19–0.25)	0.30 (0.27–0.33)	0.37 (0.33–0.42)	0.50 (0.43–0.59)
CumHDL, median (IQR), mmol/L	1.49 (1.33–1.71)	1.49 (1.33–1.71)	1.45 (1.27–1.65)	1.72 (1.52–1.95)	1.53 (1.39–1.73)	1.44 (1.30–1.59)	1.34 (1.18–1.48)
Age, mean (SD), years	52.8 ± 11.9	52.7 ± 11.9	59.2 ± 10.1	52.7 ± 11.7	53.1 ± 11.9	52.9 ± 12.0	52.3 ± 11.9
Male, No. (%)	37,692 (77.7)	37,172 (77.5)	520 (90.8)	7897 (65.1)	9211 (75.9)	10,022 (82.6)	10,562 (87.1)
BMI, mean (SD), kg/m^2^	25.1 ± 3.3	25.1 ± 3.3	26.0 ± 3.3	24.2 ± 3.2	24.9 ± 3.3	25.4 ± 3.3	25.8 ± 3.4
MHR, median (IQR)	0.22 (0.15–0.32)	0.22 (0.15–0.32)	0.26 (0.17–0.37)	0.14 (0.10–0.18)	0.19 (0.15–0.26)	0.26 (0.19–0.34)	0.35 (0.25–0.48)
Monocyte count, median (IQR), 10^9^/L	0.30 (0.20–0.41)	0.30 (0.20–0.41)	0.39 (0.30–0.50)	0.21 (0.20–0.30)	0.30 (0.20–0.40)	0.40 (0.30–0.50)	0.42 (0.30–0.60)
SBP, mean (SD), mm Hg	130.49 ± 18.95	130.4 ± 18.9	140.3 ± 20.1	128.0 ± 19.0	130.2 ± 18.9	131.4 ± 18.8	132.4 ± 18.8
DBP, median (IQR), mm Hg	80.7 (80.0–90.0)	80.7 (80.0–90.0)	88.0 (80.0–93.3)	80.0 (76.7–90.0)	80.7 (80.0–90.0)	82.0 (80.0–90.0)	83.3 (80.0–90.0)
HDL-C, median (IQR), mmol/L	1.49 (1.24–1.82)	1.49 (1.24–1.82)	1.38 (1.15–1.69)	1.78 (1.19–2.10)	1.54 (1.30–1.85)	1.40 (1.20–1.68)	1.29 (1.09–1.52)
LDL-C, mean (SD), mmol/L	2.59 ± 0.81	2.59 ± 0.80	2.75 ± 0.95	2.61 ± 0.81	2.61 ± 0.81	2.59 ± 0.80	2.54 ± 0.80
TC, mean (SD), mmol/L	4.99 ± 1.00	4.98 ± 1.00	5.32 ± 1.06	5.14 ± 1.07	5.02 ± 1.00	4.94 ± 0.96	4.84 ± 0.96
TG, median (IQR), mmol/L	1.27 (0.90–1.90)	1.27 (0.90–1.89)	1.41 (1.04–2.21)	1.13 (0.80–1.63)	1.24 (0.89–1.80)	1.33 (0.96–1.99)	1.41 (1.00–2.19)
HsCRP, median (IQR), mg/L	1.05 (0.50–2.56)	1.04 (0.50–2.53)	1.67 (0.80–3.80)	0.90 (0.49–1.88)	1.02 (0.50–2.41)	1.15 (0.48–2.80)	1.30 (0.50–3.20)
*Education, No. (%)*							
Less than high school	37,095 (76.4)	36,601 (76.33)	492 (85.86)	8747 (72.1)	9169 (75.6)	9537 (78.2)	9640 (79.5)
High school and above	11,427 (23.6)	11,348 (23.67)	81 (14.14)	3383 (27.9)	2962 (24.4)	2593 (21.4)	2491 (20.5)
Current drinker, No. (%)	16,823 (34.7)	16,663 (34.8)	181 (31.6)	4037 (33.3)	4208 (34.7)	4333 (35.8)	4266 (35.2)
*Smoking habits, No. (%)*							
Never smoker	30,046 (61.9)	29,735 (62.0)	312 (54.5)	8396 (69.2)	7741 (63.8)	7288 (60.1)	6622 (54.6)
Ever smoker	2115 (4.4)	2084 (4.4)	30 (5.2)	455 (3.8)	508 (4.2)	578 (4.8)	573 (4.7)
Current smoker	16,361 (33.7)	16,130 (33.6)	231 (40.3)	3279 (27.0)	3882 (32.0)	4264 (35.2)	4936 (40.7)
*Physical activities, No. (%)*							
Infrequent	16,407 (33.8)	16,218 (33.8)	189 (33.0)	4780 (39.4)	4225 (34.8)	3861 (31.8)	3541 (29.2)
Occasional	25,169 (51.9)	24,876 (51.9)	293 (51.1)	5366 (44.2)	6109 (50.4)	6568 (54.2)	7126 (58.7)
Frequent	6946 (14.3)	6855 (14.3)	91 (15.9)	1984 (16.4)	1797 (14.8)	1701 (14.0)	1464 (12.1))
Family history of CVD, No. (%)	6992 (14.4)	6915 (14.4)	77 (13.4)	2093 (17.3)	1821 (15.0)	1522 (12.6)	1556 (12.8)
Diabetes mellitus, No. (%)	7082 (14.6)	6934 (14.5)	148 (25.8)	1494 (12.3)	1721 (14.2)	1873 (15.4)	1994 (16.4)
Dyslipidemia, No. (%)	13,905 (28.7)	13,670 (28.5)	235 (41.0)	2885 (23.8)	3216 (26.5)	3521 (29.0)	4283 (35.3)
*Medication use, No. (%)*							
Antihypertensives	2771 (5.7)	2706 (5.6)	65 (11.3)	695 (5.7)	708 (5.8)	655 (5.4)	713 (5.9)
Anti-diabetes	2388 (4.9)	2332 (4.9)	56 (9.8)	503 (4.2)	591 (4.9)	635 (5.2)	659 (5.4)
Lipid-lowering drugs	688 (1.4)	677 (1.4)	11 (1.9)	163 (1.3)	174 (1.4)	165 (1.4)	186 (1.5)

BMI: body mass index; CumMHR: time-averaged cumulative monocyte-to-high-density lipoprotein ratio; CumMON: time-averaged cumulative monocyte count; CumHDL: time-averaged cumulative high-density lipoprotein cholesterol; CVD: cardiovascular disease; hsCRP: high-sensitivity C-reactive protein; HDL-C: high-density lipoprotein cholesterol; LDL-C: low-density lipoprotein cholesterol; MHR: monocyte-to-high-density lipoprotein cholesterol ratio; TC: total cholesterol; TG: triglyceride; SBP: systolic blood pressure; DBP: diastolic blood pressure

### CumMON, cumHDL, and CumMHR-associated risks of incident myocardial infarction in the entire cohort

**Fig. 1:**
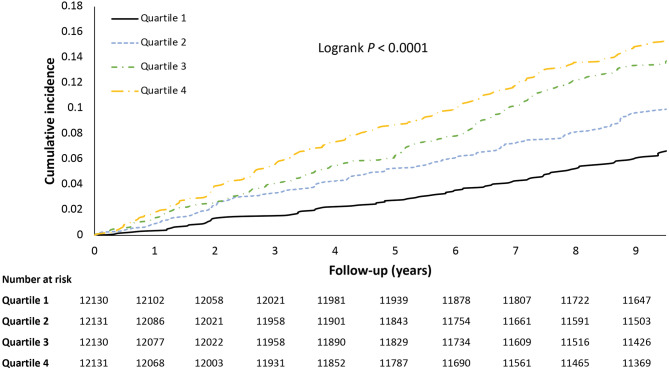
Cumulative incidence of myocardial infarction across CumMHR quartiles in the entire population. Quartile 1: Cumulative MHR quartile 1; Quartile 2: Cumulative MHR quartile 2; Quartile 3: Cumulative MHR quartile 3; Quartile 4: Cumulative MHR quartile 4

Among 48,522 participants free of CVD, 573 had MI during a median of 10.06 years of follow-up. Both CumMON and CumHDL were dose-dependently associated with incident MI. After adjusting for potential confounders, a per-SD increase in CumMON was associated with an 18% increase in the risk of incident MI (1.18, 95% CI 1.08–1.28), whereas a per-SD decrease in CumHDL was associated with a 14% decrease in MI risk (0.86, 95% CI 0.79–0.93) (both *P*-trends: <0.001). When addressing their ratio and incident MI, a more prominent risk upon CumMHR exposure was observed; a per-SD increase in CumMHR was associated with a 27% increase in the risk of incident MI (1.27, 95% CI 1.16–1.38) (Table 2). Compared to the lowest quartile of CumMHR, Quartiles 2, 3 and 4 had aHRs of 1.40 (95% CI 1.06–1.86), 1.77 (95% CI 1.35–2.32) and 1.97 (95% CI 1.51–2.58) (*p*-trend < 0.001), respectively. This association remained significant, albeit with slight attenuation after additional adjustment for total leukocyte counts. When both separate components of the MHR were included in the multivariable model, a per-SD increase in the CumMHR was associated with a greater risk (1.79, 95% CI 0.22–1.61) (*P* = 0.003). Figure 1 displays the significant difference in the cumulative MI incidence across CumMHR quartiles. A relatively weak but significant association was observed when the MHR was tested as a cross-sectional pattern; each 1-SD increase in the baseline MHR was associated with an aHR of 1.19 (95% CI 1.09–1.29) for incident MI (Supplementary Table 3).

**Table 2:** The risk of incident myocardial infarction upon exposure to cummon, CumHDL or cummhr

	Exposure to CumMON, CumHDL or CumMHR, HRs (95% CIs)				*P*-trend	Per SD
	Quartile 1	Quartile 2	Quartile 3	Quartile 4		
*Exposure to CumMON*						
Event/Total	78/12,131	151/12,130	162/12,131	182/12,130		
Incidence rate	6.4	12.5	13.4	15.1		
Unadjusted model	Reference	1.94 (1.48–2.55)	2.08 (1.59–2.73)	2.34 (1.80–3.05)	< 0.0001	1.29 (1.19–1.39)
Multivariable model	Reference	1.64 (1.25–2.16)	1.60 (1.22–2.10)	1.75 (1.34–2.29)	0.0004	1.18 (1.08–1.28)
*Exposure to CumHDL*						
Event/Total	185/12,130	134/12,131	130/12,130	124/12,131		
Incidence rate	15.4	11.1	10.7	10.3		
Unadjusted model	Reference	0.72 (0.58–0.90)	0.70 (0.56–0.87)	0.67 (0.53–0.84)	0.0004	0.86 (0.79–0.93)
Multivariable model	Reference	0.83 (0.66–1.04)	0.76 (0.61–0.96)	0.67 (0.53–0.85)	0.0008	0.86 (0.79–0.93)
*Exposure to CumMHR*						
Event/Total	80/12,130	127/12,131	170/12,130	196/12,131		
Incidence rate	6.6	10.5	14.1	16.3		
Unadjusted model	Reference	1.59 (1.20–2.10)	2.13 (1.63–2.78)	2.46 (1.90–3.19)	< 0.0001	1.35 (1.25–1.46)
Multivariable model	Reference	1.40 (1.06–1.86)	1.77 (1.35–2.32)	1.97 (1.51–2.58)	< 0.0001	1.27 (1.16–1.38)
Multivariable model + log(leukocyte count)	Reference	1.34 (1.01–1.79)	1.65 (1.25–2.18)	1.77 (1.33–2.37)	< 0.0001	1.22 (1.11–1.34)
Multivariable model + log(CumMON) + log(CumHDL)	Reference	1.34 (0.95–1.87)	1.63 (1.08–2.47)	1.73 (0.98–3.06)	0.0518	1.79 (1.22–2.61)

The multivariable-adjusted model included sex (male or female), age (continuous), BMI (continuous), smoking habits (never, ever, current), alcohol consumption (yes or no), education level (less than high school, high school and above), physical activity (infrequent, occasional, frequent), family history of CVD (yes or no), TC (continuous), SBP (continuous), diabetes status (yes or no), antihypertensive use (yes or no), antidiabetic use (yes or no), lipid-lowering drugs (yes or no) and loghsCRP (continuous)

Per SD: Risk per SD increase in log (CumMON) (0.164), log (CumHDL) (0.088) or log (CumMHR) (0.194)

The incidence rate is per 1,0000 person-years

Abbreviations are as in Table 1

### CumMHR-associated risk heterogeneity across LDL-C subgroups

**Fig. 2:**
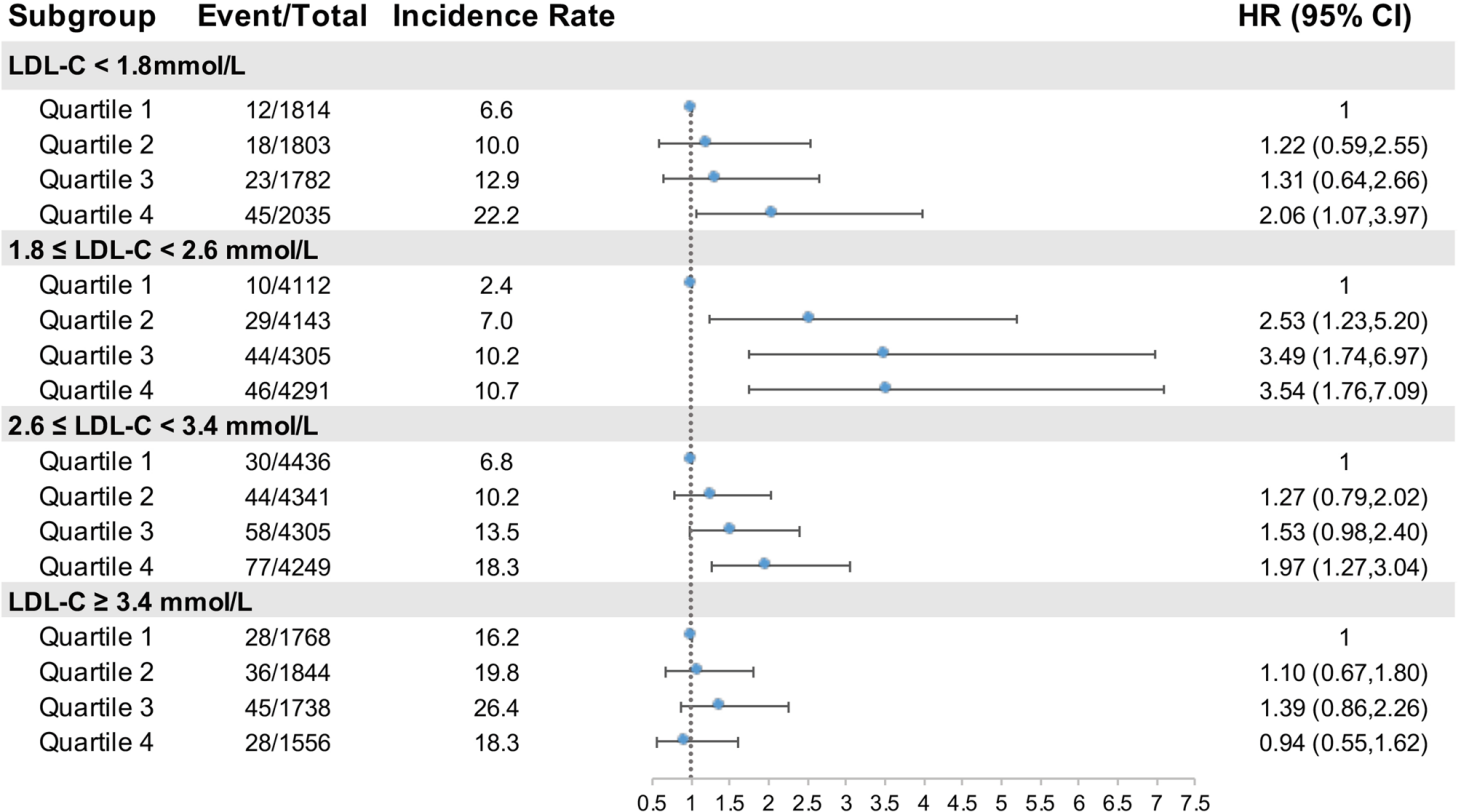
Association between the CumMHR and incident myocardial infarction stratified by LDL-C level. *P-*interaction: CumMHR quartiles*LDL-C (< 1.8, 1.8 to 2.6, 2.6 to 3.4 or ≥ 3.4 mmol/L) = 0.0574. Covariables in the multivariable-adjusted model included sex (male or female, except for sex-stratified analysis), age (continuous, except for age-stratified analysis), BMI (continuous), smoking habits (never, ever, current), alcohol consumption (yes or no), education level (less than high school, high school and above), physical activity (infrequent, occasional, frequent), family history of CVD (yes or no), SBP (continuous), diabetes status (yes or no), antihypertensives (yes or no), antidiabetics (yes or no), lipid-lowering drugs (yes or no) and loghsCRP (continuous). The incidence rate is per 1,0000 person-years. Abbreviations are as in Table 1

To test whether the CumMHR could account for the residual risk of MI beyond LDL-C, we further investigated the risk heterogeneities across different subgroups of LDL-C (Supplementary eTables 4–6). We first stratified participants by an LDL-C cutoff of 2.6 mmol/L (100 mg/L). A lower MI incidence but greater CumMHR-associated risk were found in individuals with LDL-C < 2.6 mmol/L; the aHRs per SD increase in log(CumMHR) were 1.39 (95% CI 1.22–1.59) vs. 1.14 (95% CI 1.02–1.28) for LDL-C < 2.6 vs. ≥ 2.6 mmol/L); however, the test for interaction was not statistically significant (*P* = 0.181). When the participants were stratified by an LDL-C cutoff of 3.4 mmol/L (132 mg/L), the association between the CumMHR and MI was significant at the LDL-C < 3.4 mmol/L stratum but not at the LDL-C ≥ 3.4 mmol/L stratum (*P*-interaction = 0.009). We additionally stratified the participants into 4 subgroups according to LDL-C cutoffs (< 1.8, 1.8 to 2.6, 2.6 to 3.4, and ≥ 3.4 mmol/L) and found significant risk heterogeneity (*P*-interaction = 0.057; Fig. 2). Notably, the LDL-C ≥ 3.4 mmol/L stratum had the highest MI incidence but had no significant risk of incident MI upon CumMHR exposure. In contrast, the 1.8 ≤ LDL-C < 2.6 mmol/L stratum had the lowest MI incidence but the highest CumMHR-associated MI risk: 2.53 (95% CI 1.23–5.20), 3.49 (95% CI 1.74–6.97) and 3.54 (95% CI 1.76–7.09) in CumMHR quartiles 2, 3 and 4, respectively, vs. quartile 1 (*p*-trend: < 0.001). The aHRs per SD increment in log (CumMHR) were 1.24 (95% CI 1.01–1.53), 1.50 (95% CI 1.27–1.79), 1.26 (95% CI 1.09–1.46) and 0.99 (95% CI 0.82–1.19) for LDL-C < 1.8, 1.8 to 2.6, 2.6 to 3.4 and ≥ 3.4 mmol/L, respectively. Strikingly, the highest CumMHR quartile in the LDL-C < 1.8 mmol/L stratum had markedly greater MI incidence and risk (2.06, 95% CI 1.07–3.97 vs. quartile 1; incidence rates per 1,0000 person-years of 22.2 vs. 6.6 for quartile 1).

### CumMHR-associated risk heterogeneity across other risk factors

**Fig. 3:**
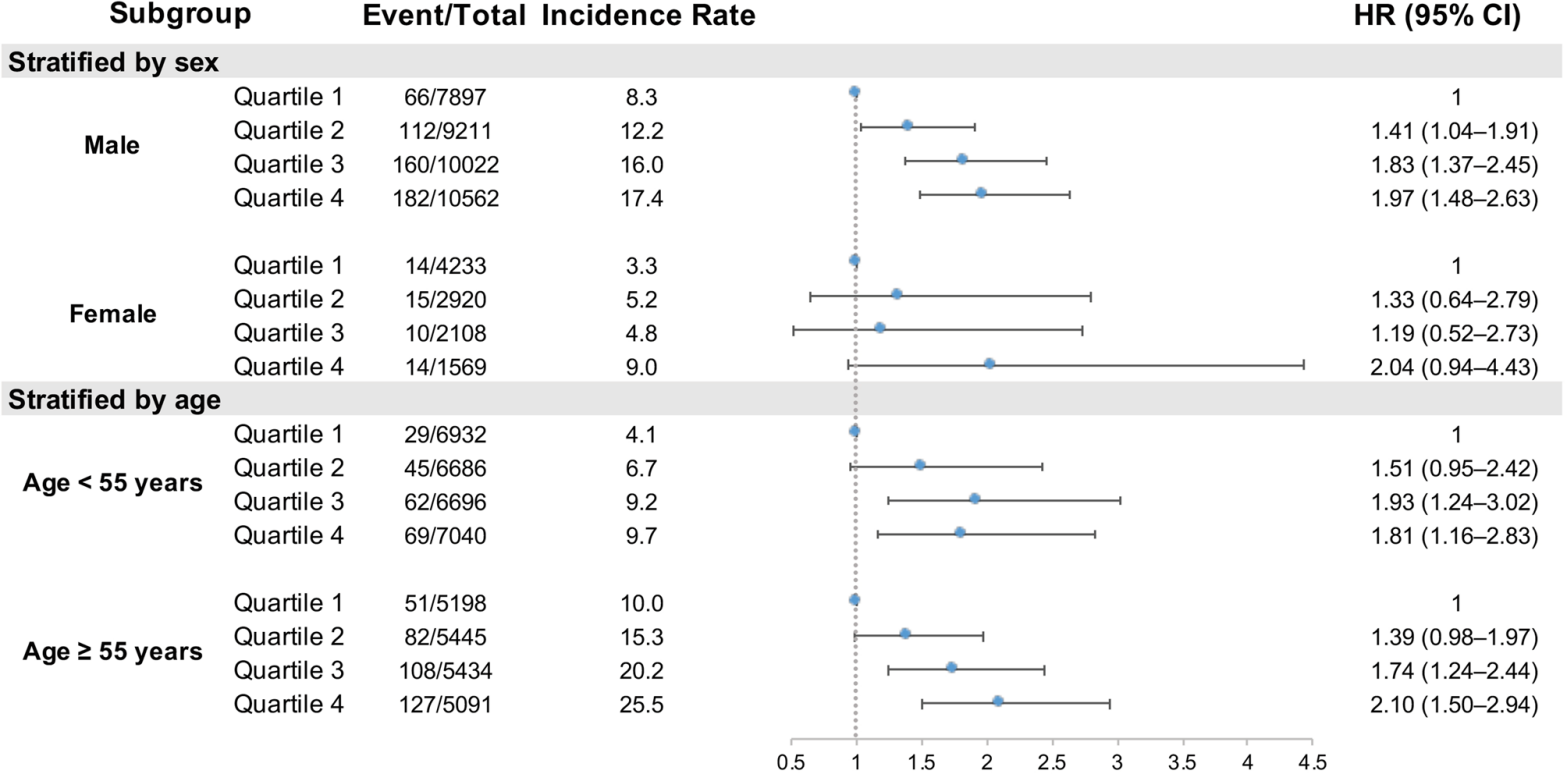
Association between the CumMHR and incident myocardial infarction stratified by sex or age. *P*-interaction: CumMHR quartiles *sex (male/female) = 0.5287; CumMHR quartile* age subgroups (< 55/≥55 years) = 0.2044. Covariables in the multivariable-adjusted model included sex (male or female, except for sex-stratified analysis), age (continuous, except for age-stratified analysis), BMI (continuous), smoking habits (never, ever, current), alcohol consumption (yes or no), education level (less than high school, high school and above), physical activity (infrequent, occasional, frequent), family history of CVD (yes or no), TC (continuous), SBP (continuous), diabetes (yes or no), antihypertensives (yes or no), antidiabetics (yes or no), lipid-lowering drugs (yes or no) and loghsCRP (continuous). The incidence rate is per 1,0000 person-years. Abbreviations are as in Table 1

**Fig. 4:**
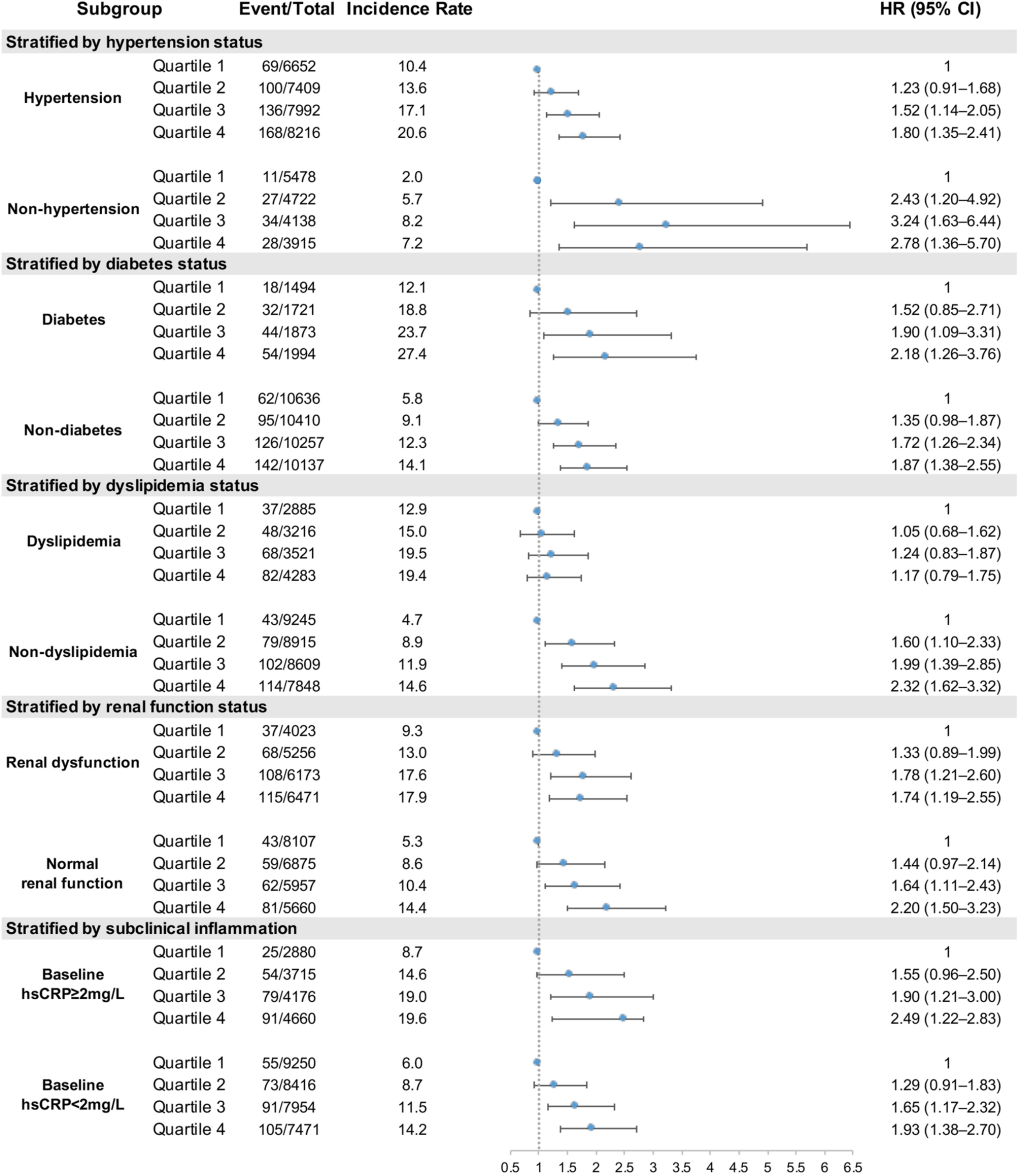
Association between the CumMHR and incident myocardial infarction stratified by prevalent disease or subclinical inflammation. *P*-interaction: CumMHR quartiles*hypertension (yes or no) = 0.1573; CumMHR quartiles*diabetes (yes or no) = 0.9947; CumMHR quartiles*dyslipidemia (yes or no) = 0.0691; CumMHR quartiles*renal dysfunction (yes or no) = 0.4947. Covariables in the multivariable-adjusted mode included sex (male or female), age (continuous), BMI (continuous), smoking habits (never, ever, current), alcohol consumption (yes or no), education level (less than high school, high school and above), physical activity (infrequent, occasional, frequent), family history of CVD (yes or no), TC (continuous, except for dyslipidemia-stratified analysis), SBP (continuous, except for hypertension-stratified analysis), diabetes status (yes or no, except for diabetes-stratified analysis), (yes or no), antidiabetics (yes or no), lipid-lowering drugs (yes or no) and loghsCRP (continuous, except for inflammation-stratified analysis). The incidence rate is per 1,0000 person-years. Abbreviations are as in Table 1

Subgroup analyses were further performed to address the heterogeneity in MI risk associated with CumMHR exposure (Figs. 3 and 4, Supplementary eTables 7–13). Male individuals tended to have higher MI incidence rates and higher CumMHR-associated risks for incident MI; each 1-SD increase was associated with an aHR of 1.28 (95% CI 1.17–1.40) for males vs. 1.18 (95% CI 0.90–1.55) for females. When examining the risk heterogeneity across age distributions, older individuals appeared to have consistently higher rates and greater risks of incident MI with exposure to CumMHR. The aHRs per 1-SD increase in log(CumMHR) were 1.33 (95% CI 1.19–1.48) vs. 1.17 (95% CI 1.01–1.34) for individuals aged ≥ 55 vs. < 55 years. Similarly, higher MI incidence rates were unsurprisingly observed among participants with prevalent morbidities than among those without morbidities, yet the CumMHR-MI association appeared to differ across different disease statuses. Despite higher MI incidences, participants with hypertension, renal dysfunction or dyslipidemia tended to have a weaker but more significant association between the CumMHR and incident MI. The aHRs per SD increase in CumMHR were 1.27 (95% CI 1.14–1.38) for hypertension vs. 1.29 (95% CI 1.05–1.57) for non-hypertension, 1.09 (95% CI 0.95–1.24) for dyslipidemia vs. 1.31 (95% CI 1.17–1.47) for non-dyslipidemia and 1.18 (95% CI 1.05–1.33) for impaired renal function vs. 1.35 (95% CI 1.19–1.53) for normal renal function. In comparison, individuals with diabetes or elevated inflammation appeared to have a consistently greater incidence and risk of MI with exposure to CumMHR. The aHRs (each 1-SD increment) for CumMHR were 1.31 (95% CI 1.11–1.55) vs. 1.25 (95% CI 1.13–1.38) for diabetes vs. non-diabetes and 1.30 (95% CI 1.13–1.48) vs. 1.24 (95% CI 1.10–1.38) for hsCRP ≥ 2 vs. < 2 mg/L. Tests for interactions between CumMHR quartiles and these risk factors were not statistically significant.

In the sensitivity analyses, similar results to the primary findings were obtained after excluding MI onset within the first year of follow-up, addressing the competitive risk of death by Fine-Gray model and repeating the analysis based on the raw data as well as adjusting only for covariates with SMD > 0.10 (Supplementary Tables 14–17).

## Discussion

In a real-life, prospective cohort in China, we found a significant dose-dependent association between the time-averaged cumulative MHR, monocyte count or HDL-C and incident MI among the general population. A graded increase in the CumMHR was positively correlated with a clustering of traditional risk factors but inversely associated with LDL-C levels. Notably, the CumMHR-MI association differed significantly across different LDL-C levels and was more prominent at low LDL-C levels. Additionally, increased CumMHR tended to heighten the risk of incident MI among individuals of male sex, old age, prevalent diabetes or subclinical inflammation.

Monocytes, as representatives of the innate immune system, play a major role in the initiation, propagation and progression of atherosclerosis from a stable to an unstable state [21, 42] as well as in outcomes post infarction [20, 22]. Studies in atherosclerotic mice have shown that bone marrow–derived circulating monocytes populate atherosclerotic lesions [42, 43]. Consistent with the significant association between elevated monocyte counts and MI found in our study, a previous study demonstrated that a high monocyte count may predict the premature occurrence of a coronary event in 3,779 French middle-aged men [44]. Additionally, converging evidence has demonstrated the prognostic significance of the total monocyte count [28] and monocyte subset [29] in patients with existing cardiac ischemic diseases. The chronically activated hematopoietic activity resulting from continuous stimulation of enriched cardiovascular risk factors may partly provide a mechanistic explanation for the unfavorable outcome of the systemic increase in circulating monocytes. Suboptimal lifestyle patterns (e.g., excess caloric intake [45, 46], deficient sleep [47] or physical inactivity [48]), a wide range of metabolic disorders and morbidities (e.g., hypertension [49, 50], hyperglycemia [14, 15], cholesterol defects [16, 17, 51, 52], renal dysfunction [7] and autoimmune diseases [9, 10]) have been demonstrated to induce a sustained increase in the circulating monocyte pool and favor thermogenesis. In contrast, increased HDL-C has a suppressive role in controlling monocyte activation, proliferation and differentiation of other progenitor cells, as well as in the formation of foam cells [53–55], thereby eliminating the proinflammatory and pro-oxidant effects induced by monocytosis and benefiting cardiovascular health [56, 57]. Indeed, compelling evidence from experiment-based studies has consistently demonstrated the suppressive effect of increasing HDL-C levels on monocytosis and atherosclerosis [11, 16–19]. Overall, the significant roles of monocytes and HDL-C, as well as their close biological interplay in the development of atherosclerotic diseases, support the utility of the MHR as a biomarker for cardiovascular risk prediction.

Notably, our findings demonstrated significant risk heterogeneity across different LDL-C levels with time-averaged exposure to MHR. Individuals with LDL-C levels between 1.8 and 2.6 mmol/L but not those with LDL-C levels < 1.8 mmol/L had the lowest incidence of MI. This pattern aligns with the “J-shaped” association between LDL-C and ischemic CVD risk reported in emerging studies [58, 59]. For example, in a cohort of over 2.4 million individuals without prior ASCVD or statin use, MI and ischemic stroke risks were lowest at LDL-C 70–99 mg/dL (≈ 1.8–2.6 mmol/L) and increased at both lower and higher LDL-C levels [58]. In our study, participants with LDL-C levels ≥ 3.4 mmol/L had a markedly greater MI incidence but a nonsignificant risk of incident MI upon CumMHR exposure. In contrast, lower MI incidences but significantly greater risks of incident MI upon CumMHR exposure were observed in the 1.8 < LDL-C ≤ 2.6 mmol/L stratum. Below this range, increased CVD risk may be attributable to comorbid conditions or reverse causation. In this optimal LDL-C range, where lipid burden is moderate, inflammatory markers such as MHR may have greater discriminative power for identifying high-risk individuals. These results support the potential of optimizing the MI risk assessment by assessing the CumMHR among individuals with low LDL-C levels. Although the full picture of monocytosis and defective cholesterol metabolism remains to be elucidated, therapeutic strategies targeting chronic imbalances in monocytes and HDL-C could afford MI-preventive benefits and therefore facilitate precision medicine.

Diabetes and chronic inflammation have been proposed to account for residual cardiovascular risks despite aggressive LDL-C lowering [3–6]. In our study, an elevated CumMHR tended to enhance both MI incidence and MI risk among individuals with diabetes or elevated inflammation, differing from the weaker association between the CumMHR and MI observed in other diseases. The notably elevated monocytosis in conjunction with decreased HDL-C levels in patients with diabetes or chronic immune diseases [7, 9–15] provided support for these findings. Additionally, in mouse models of hyperglycemia, increasing HDL-C levels by infusing apolipoprotein AI [18] or mir-33 [19] could overcome the poor regression of atherosclerotic plaques after LDL-C-lowering treatment, with mechanisms involving HDL-C-induced inhibition of hyperglycemia-enhanced proliferation of monocytes. Furthermore, infusion of reconstituted HDL (rHDL) in the RA setting induces ATP-binding cassette transporter expression and inhibits monocytosis, thereby reversing inflammation-induced changes in lesion development and reducing residual cardiovascular risk [11]. Indeed, therapeutics directed at preventing abnormal hematopoiesis, including anti-inflammatory agents and drugs that suppress monocytosis and rHDL infusions, have been suggested to be important supplements to traditional approaches for lowering LDL-C levels to further prevent atherothrombosis [8, 20].

### Clinical implications

Lowering LDL-C levels is a priority for the primary and secondary prevention of atherosclerosis and its complications. Our study provided data-based epidemiological evidence for the significant association between the CumMHR and residual cardiovascular risk in individuals with low LDL-C levels. In view of our findings, longitudinal tracing of MHR changes may aid in residual risk prediction and management among individuals with low LDL-C levels, male sex, old age, diabetes or elevated inflammation. Targeting chronic imbalances in monocyte counts and HDL-C levels may therefore represent a novel approach for personalizing treatment plans and guiding more intensive disease management strategies to further reduce MI risk.

This study has several strengths. This is the first large-scale population-based study to longitudinally investigate the association between chronic imbalances in circulating monocyte and HDL-C levels and future incident MI among the general population. Additionally, the circulating monocyte pool highly oscillates [60] because it is easily influenced by diverse lifestyle factors [15, 45–48]; therefore, the present study used time-averaged exposure derived from repeatedly measured data, which would provide a more reliable estimate of the results. Other merits of the study included the large sample size, the long follow-up period and the use of high-quality and longitudinally measured data from the prospective, real-life cohort.

This study has several limitations. First, the analysis was conducted based on data from community-dwelling Han Chinese adults in northern China, which may have limited the generalizability of the findings to the entire country and/or other ethnic groups. Second, the study population was predominantly male, which may further constrain extrapolation; however, our sex-stratified analyses partially address this limitation. Regional dietary patterns (e.g., higher salt intake), genetic backgrounds, stress and socioeconomic factors may influence monocyte activity and lipid metabolism, thereby modifying the MHR–MI association. Further validation in diverse, multi-ethnic cohorts, including women and individuals from different geographic and socioeconomic strata, is warranted to confirm the external applicability of these findings. Third, we failed to discriminate chronic autoimmune diseases (e.g., RA and SLE) among the study population; however, the subgroup analysis across different levels of inflammation in our study may have partly helped to interpret the prominent cardiovascular risks in chronic inflammatory settings. Fourth, excluding participants with prior CVD and those without completed exposure data between 2006/2007 and 2010/2011, while intentional and aligned with our study aim, likely produced a healthier analytic cohort and may affect the generalizability of the findings. In observational cohort studies, such selection based on study objectives and exposure definitions is common and acceptable. Furthermore, residual confounding from unmeasured factors (e.g., diet, stress, other inflammatory biomarkers, anti-inflammatory treatment) cannot be excluded, but we adjusted for a comprehensive set of guideline-recommended cardiovascular risk factors likely capturing major known sources of confounding.

## Conclusions

Chronic imbalances in monocyte and HDL-C levels are significantly associated with incident MI among individuals with low LDL-C levels. Longitudinally tracing MHR changes and targeting elevated MHR may help to optimize risk assessment and management of MI and facilitate precise medicine.

## Supplementary Information

Below is the link to the electronic supplementary material.

Supplementary Material 1

## Data Availability

The data sets analysed during the current study are not publicly available due to legal and ethical restraints. Data are available from the corresponding author on reasonable request.

## Abbreviations

aHRs: Adjusted hazard ratios
BMI: Body mass index
CumHDL: Time-averaged cumulative high-density lipoprotein cholesterol
CumMON: Time-averaged cumulative monocyte count
CumMHR: Time-averaged Cumulative monocyte-to-HDL-C ratio
CVD: Cardiovascular disease
HDL-C: High-density lipoprotein cholesterol
hsCRP: High-sensitivity C-reactive protein
IQRs: Interquartile ranges
LDL-C: Low-density lipoprotein cholesterol
MI: Myocardial infarction
RCR: Residual cardiovascular risk
rHDL: Reconstituted HDL
SBP: Systolic blood pressure
SD: Standard deviation
TC: Total cholesterol
