# Correction: Imbalances in circulating monocyte and high-density lipoprotein cholesterol exacerbates the residual risk of incident myocardial infarction beyond LDL-C: a real-life, prospective cohort study

**DOI:** 10.1186/s12967-026-07834-7

**Published:** 2026-02-11

**Authors:** Dan Wu, Yulong Lan, Xiong Ding, Lois Balmer, Xingang Li, Wei Wang, Shouling Wu, Youren Chen

**Affiliations:** 1 Department of Cardiology, Second Affiliated Hospital of Shantou University Medical College, 69 Dongxia North Road, Jinping District, Shantou, 515041 China; 2 Centre for Precision Health, School of Medical and Health Sciences, Edith Cowan University, Room 521, Building 21/270 Joondalup Drive, Perth, WA 6027 Australia; 3 School of Public Health, Wuhan University, Wuhan, 430072 China; 4 Clinical Research Centre, First Affiliated Hospital of Shantou University Medical College, Shantou, 515041 China; 5 Beijing Key Laboratory of Clinical Epidemiology, School of Public Health, Capital Medical University, Beijing, 100069 China; 6 School of Public Health, Shandong First Medical University & Shandong Academy of Medical Sciences, Tai’an, 271016 China; 7 Department of Cardiology, Kailuan General Hospital, 57 Xinhua East Road, Lubei District, Tangshan, 063000 China

**Correction: Journal of Translational Medicine (2025) 23:1433**

10.1186/s12967-025-07028-7

The equation following the sentence “divided by the total exposure period:”

$$\begin{aligned}\:[\mathrm{t}\mathrm{i}\mathrm{m}\mathrm{e}\:1-2&{*}(\mathrm{V}\mathrm{a}\mathrm{l}\mathrm{u}\mathrm{e}\:1+\mathrm{V}\mathrm{a}\mathrm{l}\mathrm{u}\mathrm{e}\:2)/2+\mathrm{t}\mathrm{i}\mathrm{m}\mathrm{e}\:2-3\cr&{*}(\mathrm{V}\mathrm{a}\mathrm{l}\mathrm{u}\mathrm{e}\:2+\mathrm{V}\mathrm{a}\mathrm{l}\mathrm{u}\mathrm{e}\:3)/2]\:\mathrm{t}\mathrm{i}\mathrm{m}\mathrm{e}\:1-3\end{aligned}$$

of this article, the division symbol “/” before “time 1 − 3” was not displayed.

This has now been corrected.

$$\begin{aligned}\:[\mathrm{t}\mathrm{i}\mathrm{m}\mathrm{e}\:1-2&{*}(\mathrm{V}\mathrm{a}\mathrm{l}\mathrm{u}\mathrm{e}\:1+\mathrm{V}\mathrm{a}\mathrm{l}\mathrm{u}\mathrm{e}\:2)/2+\mathrm{t}\mathrm{i}\mathrm{m}\mathrm{e}\:2-3\cr&{*}(\mathrm{V}\mathrm{a}\mathrm{l}\mathrm{u}\mathrm{e}\:2+\mathrm{V}\mathrm{a}\mathrm{l}\mathrm{u}\mathrm{e}\:3)/2]\:/\:\mathrm{t}\mathrm{i}\mathrm{m}\mathrm{e}\:1-3\end{aligned}$$

The original article has been updated accordingly.

