# Supplementary material for: Imbalances in circulating monocyte and high-density lipoprotein cholesterol exacerbates the residual risk of incident myocardial infarction beyond LDL-C: a real-life, prospective cohort study
Source: J Transl Med. 2025 Dec 30;23:1433. doi: 10.1186/s12967-025-07028-7 (PMC12754891; doi:10.1186/s12967-025-07028-7)
Supplement: Supplementary file 1 — Supplementary Material 1 [file 12967_2025_7028_MOESM1_ESM.docx]

**Supplementary Materials**

**Table of Contents**

[**eTable 1.** Baseline difference between included and excluded participants 3](#_Toc205665544)

[**eTable 3.** Baseline MHR-associated risk of incident MI in the entire cohort 7](#_Toc205665545)

[**eTable 4.** CumMHR-associated risk of incident MI according to baseline LDL-C levels (<2.6/≥2.6 mmol/L) 8](#_Toc205665546)

[**eTable 5.** CumMHR-associated risk of incident MI according to LDL-C levels (<3.4/≥3.4 mmol/L) 9](#_Toc205665547)

[**eTable 6.** CumMHR-associated risk of incident MI according to LDL-C levels (<1.8, 1.8 to 2.6,2.6 to 3.4 or ≥3.4 mmol/L) 10](#_Toc205665548)

[**eTable 7.** CumMHR-associated risk of incident MI according to sex (male/female) 12](#_Toc205665549)

[**eTable 8.** CumMHR-associated risk of incident MI according to age subgroups (<55/≥55 years) 13](#_Toc205665550)

[**eTable 9.** CumMHR-associated risk of incident MI according to prevalent hypertension (yes or no) 14](#_Toc205665551)

[**eTable 10.** CumMHR-associated risk of incident MI according to prevalent diabetes (yes or no) 15](#_Toc205665552)

[**eTable 11.** CumMHR-associated risk of incident MI according to prevalent dyslipidemia (yes or no) 16](#_Toc205665553)

[**eTable 12.** CumMHR-associated risk of incident MI according to renal dysfunction (yes or no) 17](#_Toc205665554)

[**eTable 13.** CumMHR-associated risk of incident MI according to subclinical inflammation (hsCRP<2/≥2mg/L) 18](#_Toc205665555)

[**eTable 14.** Reverse analysis of CumMHR-associated risk of incident MI in the entire cohort (520/48469) 19](#_Toc205665556)

[**eTable 15.** Fine-Gray model of CumMHR-associated risk of incident MI in the entire cohort 20](#_Toc205665557)

[**eTable 16.** CumMHR-associated risk of incident MI in the entire cohort on unimputed data (560/47693) 21](#_Toc205665558)

[**eFigure 1.** Flowchart of the study participants 23](#_Toc205665559)

[**eFigure 2.** Study design of the present analysis 24](#_Toc205665560)

[**eFigure 3.** Missing rates of covariates 25](#_Toc205665561)

[**eFigure 4.** Missing pattern of covariates 26](#_Toc205665562)

[**Supplementary methods** 27](#_Toc205665563)

# **eTable 1.** Baseline difference between included and excluded participants

|  | **Included** | **Excluded** | ***P*-difference** | **Missing n (Included)** | **Missing n (Excluded)** |
| --- | --- | --- | --- | --- | --- |
| Age, mean (SD), years | 52.8±11.9 | 55.7±12.4 | <0.0001 | 0 | 0 |
| Male, No. (%) | 37692 (77.7) | 43173 (81.5) | <0.001 | 0 | 0 |
| Education, No. (%) |  |  | <0.001 | 5 | 1035 |
| Less than high school | 37090 (76.4) | 42905(81.0) |  |  |  |
| High school and above | 11427 (23.6) | 9048 (17.1) |  |  |  |
| Prevailing CVD, No. (%) | 0 | 7442 (14.0) | <0.001 | 0 | 0 |
| BMI, mean (SD), kg/m^2^ | 25.1±3.3 | 25.2±3.4 | <0.001 | 145 | 33080 |
| MHR, median (IQR) | 0.22 (0.15–0.32) | 0.23 (0.15–0.33) | <0.001 | 0 | 33381 |
| Monocyte count, median (IQR), 10^9^/L | 0.30 (0.20–0.41) | 0.30 (0.20–0.41) | 0.011 | 0 | 33291 |
| SBP, mean (SD), mm Hg | 130.49±18.95 | 135.20±21.42 | <0.001 | 81 | 33037 |
| DBP, median (IQR), mm Hg | 80.7 (80.0–90.0) | 84.0 (80.0–91.3) | <0.001 | 92 | 33045 |
| HDL-C, median (IQR), mmol/L | 1.49 (1.24–1.82) | 1.44 (1.25–1.75) | <0.001 | 0 | 33313 |
| LDL-C, mean (SD), mmol/L | 2.59±0.81 | 2.62±0.78 | <0.001 | 50 | 33325 |
| TC, mean (SD), mmol/L | 4.99±1.00 | 4.96±1.03 | 0.003 | 32 | 33317 |
| TG, median (IQR), mmol/L | 1.27 (0.90–1.90) | 1.27 (0.95–1.89) | 0.002 | 52 | 33320 |
| HsCRP, median (IQR), mg/L | 1.05 (0.50–2.56) | 1.00 (0.41–2.33) | <0.001 | 607 | 33503 |
| Creatine, median (IQR), mg/L | 80.24±20.94 | 83.36±21.44 | <0.001 | 43 | 33325 |
| Current drinker, No. (%) | 16843 (34.7) | 6312 (31.3) | <0.001 | 5 | 32821 |
| Smoking habits, No. (%) |  |  | <0.001 | 5 | 32818 |
| Never smoker | 30043 (61.9) | 12648 (62.7) |  |  |  |
| Ever smoker | 2114 (4.4) | 1149 (5.7) |  |  |  |
| Current smoker | 16360 (33.7) | 6373 (31.6) |  |  |  |
| Physical activities, No. (%) |  |  | <0.001 | 3 | 32768 |
| Infrequent | 16404 (33.8) | 5622 (27.8) |  |  |  |
| Occasional | 25169 (51.9) | 11404 (56.4) |  |  |  |
| Frequent | 6946 (14.3) | 3194 (15.8) |  |  |  |
| Medication use, No. (%) |  |  |  |  |  |
| Antihypertensives | 2771 (5.7) | 2143 (10.6) | <0.001 | 0 | 32764 |
| Anti-diabetes | 2388 (4.9) | 1267 (6.3) | <0.001 | 0 | 32764 |
| Lipid-lowering drugs | 688 (1.4) | 568 (2.8) | <0.001 | 0 | 32764 |

Captions: BMI: body mass index; CVD: cardiovascular disease; hsCRP: high-sensitivity C-reactive protein; HDL-C: high-density lipoprotein cholesterol; LDL-C: low-density lipoprotein cholesterol; MHR: monocyte-to-high-density lipoprotein cholesterol ratio; TC: total cholesterol; TG: triglyceride; SBP: systolic blood pressure; DBP: diastolic blood pressure.

# **eTable 2** Standardized mean differences of covariates between individuals with and with MI onset

|  | **Non-MI**  **(47,949)** | **MI**  **(n=573)** | **Standardized mean differences** |
| --- | --- | --- | --- |
| Age, mean (SD), years | 52.7±11.9 | 59.2±10.1 | 0.546 |
| Male, No. (%) | 37172 (77.5) | 520 (90.8) | 0.371 |
| BMI, mean (SD), kg/m^2^ | 25.1±3.3 | 26.0±3.3 | 0.273 |
| SBP, mean (SD), mm Hg | 130.4±18.9 | 140.3±20.1 | 0.524 |
| LDL-C, mean (SD), mmol/L | 2.59±0.80 | 2.75±0.95 | 0.199 |
| TC, mean (SD), mmol/L | 4.98±1.00 | 5.32±1.06 | 0.340 |
| TG, median (IQR), mmol/L | 1.27 (0.90–1.89) | 1.41 (1.04–2.21) | 0.175 |
| Log(hsCRP), mean (SD), mg/L | -0.09±0.781 | 0.09±0.76 | 0.234 |
| Log(Leukocyte), mean (SD), | 0.79±0.11 | 0.82±0.10 | 0.313 |
| Education, No. (%) |  |  | 0.255 |
| Less than high school | 36601 (76.33) | 492 (85.86) |  |
| High school and above | 11348 (23.67) | 81 (14.14) |  |
| Current drinker, No. (%) | 16663 (34.8) | 181 (31.6) | 0.067 |
| Smoking habits, No. (%) |  |  | 0.214 |
| Never smoker | 29735 (62.0) | 312 (54.5) |  |
| Ever smoker | 2084 (4.4) | 30 (5.2) |  |
| Current smoker | 16130 (33.6) | 231 (40.3) |  |
| Physical activities, No. (%) |  |  | 0.051 |
| Infrequent | 16218 (33.8) | 189 (33.0) |  |
| Occasional | 24876 (51.9) | 293 (51.1) |  |
| Frequent | 6855 (14.3) | 91 (15.9) |  |
| Family history of CVD,  No. (%) | 6915 (14.4) | 77 (13.4) | 0.029 |
| Diabetes mellitus, No. (%) | 6934 (14.5) | 148 (25.8) | 0.322 |
| Dyslipidemia, No. (%) | 13670 (28.5) | 235 (41.0) | 0.277 |
| Medication use, No. (%) |  |  |  |
| Antihypertensives | 2706 (5.6) | 65 (11.3) | 0.246 |
| Anti-diabetes | 2332 (4.9) | 56 (9.8) | 0.227 |
| Lipid-lowering drugs | 677 (1.4) | 11 (1.9) | 0.043 |

Captions: BMI: body mass index; CVD: cardiovascular disease; hsCRP: high-sensitivity C-reactive protein; LDL-C: low-density lipoprotein cholesterol; MI: myocardial infarction; MHR: monocyte-to-high-density lipoprotein cholesterol ratio; TC: total cholesterol; TG: triglyceride; SBP: systolic blood pressure.

# **eTable 3.** Baseline MHR-associated risk of incident MI in the entire cohort

|  | **Baseline MHR, HRs (Cls)** | | | | ***P*-trend** | **Per SD** |
| --- | --- | --- | --- | --- | --- | --- |
|  | **Quartile 1** | **Quartile 2** | **Quartile 3** | **Quartile 4** |  |  |
| Event/Total | 92/12143 | 140/12100 | 137/12125 | 204/12154 |  |  |
| Incidence rate | 7.6 | 11.7 | 11.4 | 16.8 |  |  |
| Unadjusted model | Reference | 1.54 (1.18–2.00) | 1.50 (1.15–1.95) | 2.22 (1.74–2.84) | <0.001 | 1.30 (1.19–1.41) |
| Multivariable model | Reference | 1.36 (1.04–1.76) | 1.22 (0.93–1.59) | 1.70 (1.34–2.18) | <0.001 | 1.17 (1.08–1.28) |
| Multivariable model + leukocyte count | Reference | 1.26 (0.97–1.65) | 1.12 (0.84–1.47) | 1.54 (1.17–2.03) | 0.006 | 1.13 (1.02–1.24) |

The multivariable model was adjusted for sex, age (continuous), BMI (continuous), smoking habits (never, ever, current), alcohol consumption (yes or no), education level (less than high school, high school and above), physical activity (infrequent, occasional, frequent), family history of CVD (yes or no), TC (continuous), SBP (continuous), diabetes (yes or no), loghsCRP (continuous), antihypertensives (yes or no), antidiabetics (yes or no), lipid-lowering drugs (yes or no).

Per SD: risk per SD increase in log (MHR) (0.258).

The incidence rate is per 1,0000 person-years.

Abbreviations: BMI: body mass index; CI: confidence interval; CVD: cardiovascular disease; hsCRP: high-sensitivity C-reactive protein; HDL-C: high-density lipoprotein cholesterol; HR: hazard ratio; LDL-C: low-density lipoprotein cholesterol; MI: myocardial infarction; MHR: monocyte-to-high-density lipoprotein cholesterol ratio; TC: total cholesterol; TG: triglyceride; SBP: systolic blood pressure; SD: standard deviation.

# **eTable 4.** CumMHR-associated risk of incident MI according to baseline LDL-C levels (<2.6/≥2.6 mmol/L)

|  | **CumMHR, HRs (95% CIs)** | | | | ***P*-trend** | **Per SD** |
| --- | --- | --- | --- | --- | --- | --- |
|  | **Quartile 1** | **Quartile 2** | **Quartile 3** | **Quartile 4** |  |  |
| **LDL-C <2.6 mmol/L (227/24285)** | | | | | | |
| Event/Total | 22/5926 | 47/5946 | 67/6087 | 91/6326 |  |  |
| Incidence rate | 3.7 | 7.9 | 11.0 | 14.4 |  |  |
| Unadjusted model | Reference | 2.13 (1.29–3.54) | 2.97 (1.84–4.81) | 3.90 (2.45–6.21) | <0.001 | 1.54 (1.36–1.74) |
| Multivariable model | Reference | 1.80 (1.08–2.99) | 2.28 (1.40–3.72) | 2.76 (1.72–4.44) | <0.001 | 1.39 (1.22–1.59) |
| **LDL-C≥2.6 mmol/L (346/24237)** | | | | | | |
| Event/Total | 58/6204 | 80/6185 | 103/6043 | 105/5805 |  |  |
| Incidence rate | 9.41 | 13.0 | 17.2 | 18.3 |  |  |
| Unadjusted model | Reference | 1.39 (0.99–1.94) | 1.83 (1.33–2.52) | 1.94 (1.41–2.68) | <0.001 | 1.25 (1.13–1.39) |
| Multivariable model | Reference | 1.18 (0.84–1.66) | 1.45 (1.05–2.02) | 1.48 (1.06–2.06) | 0.010 | 1.14 (1.02–1.28) |
| *P*-interaction: CumMHR quartiles*LDL-C (<2.6 or ≥2.6 mmol/L) = 0.181 | | | | | | |

Model 1: adjusted for sex, age (continuous), BMI (continuous), smoking habits (never, ever, current), alcohol consumption (yes or no), education level (less than high school, high school and above), physical activity (infrequent, occasional, frequent), family history of CVD (yes or no).

Model 2: further adjusted for loghsCRP (continuous), SBP (continuous), diabetes (yes or no), antihypertensives (yes or no), antidiabetics (yes or no), lipid-lowering drugs (yes or no).

Per SD: risk per SD increase in log (CumMHR) (0.194).

The incidence rate is per 1,0000 person-years.

Abbreviations: CumMHR, time-averaged cumulative monocyte-to-high density lipoprotein ratio; others were as in eTable 1.

# **eTable 5.** CumMHR-associated risk of incident MI according to LDL-C levels (<3.4/≥3.4 mmol/L)

|  | **CumMHR, HRs (95% CIs)** | | | | ***P*-trend** | **Per SD** |
| --- | --- | --- | --- | --- | --- | --- |
|  | **Quartile 1** | **Quartile 2** | **Quartile 3** | **Quartile 4** |  |  |
| **LDL-C <3.4 mmol/L (436/41616)** | | | | | | |
| Event/Total | 52/10362 | 91/10287 | 125/10392 | 168/10575 |  |  |
| Incidence rate | 5.0 | 8.9 | 12.1 | 16.0 |  |  |
| Unadjusted model | Reference | 1.77 (1.26–2.48) | 2.40 (1.74–3.32) | 3.19 (2.34–4.35) | <0.001 | 1.46 (1.34–1.60) |
| Multivariable model | Reference | 1.49 (1.06–2.10) | 1.85 (1.33–2.57) | 2.28 (1.65–3.13) | <0.001 | 1.32 (1.20–1.46) |
| **LDL-C ≥3.4 mmol/L (137/6906)** | | | | | | |
| Event/Total | 28/1768 | 36/1844 | 45/1738 | 28/1556 |  |  |
| Incidence rate | 16.22 | 19.8 | 26.4 | 18.3 |  |  |
| Unadjusted model | Reference | 1.24 (0.75–2.02) | 1.64 (1.03–2.63) | 1.14 (0.67–1.92) | 0.351 | 1.06 (0.90–1.26) |
| Multivariable model | Reference | 1.10 (0.67–1.80) | 1.39 (0.86–2.26) | 0.94 (0.55–1.62) | 0.897 | 0.99 (0.82–1.19) |
| *P*-interaction: CumMHR quartiles*LDL-C (<3.4 or ≥3.4 mmol/L) = 0.009 | | | | | | |

The multivariable model was adjusted for sex, age (continuous), BMI (continuous), smoking habits (never, ever, current), alcohol consumption (yes or no), education level (less than high school, high school and above), physical activity (infrequent, occasional, frequent), family history of CVD (yes or no).

Model 2: further adjusted for loghsCRP (continuous), SBP (continuous), diabetes (yes or no), antihypertensives (yes or no), antidiabetics (yes or no), lipid-lowering drugs (yes or no).

Per SD: risk per SD increase in log (CumMHR) (0.194).

The incidence rate is per 1,0000 person-years.

Abbreviations: CumMHR, time-averaged cumulative monocyte-to-high density lipoprotein ratio; others were as in eTable 1.

# **eTable 6.** CumMHR-associated risk of incident MI according to LDL-C levels (<1.8, 1.8 to 2.6,2.6 to 3.4 or ≥3.4 mmol/L)

|  | **CumMHR, HRs (95% CIs)** | | | | ***P*-trend** | **Per SD** |
| --- | --- | --- | --- | --- | --- | --- |
|  | **Quartile 1** | **Quartile 2** | **Quartile 3** | **Quartile 4** |  |  |
| **LDL-C <1.8 mmol/L (98/7434)** | | | | | | |
| Event/Total | 12/1814 | 18/1803 | 23/1782 | 45/2035 |  |  |
| Incidence rate | 6.6 | 10.0 | 12.9 | 22.2 |  |  |
| Unadjusted model | Reference | 1.51 (0.73–3.13) | 1.94 (0.97–3.91) | 3.34 (1.77–6.31) | <0.001 | 1.43 (1.19–1.72) |
| Multivariable model | Reference | 1.22 (0.59–2.55) | 1.31 (0.64–2.66) | 2.06 (1.07–3.97) | 0.014 | 1.24 (1.01–1.53) |
| **1.8≤LDL-C****<2.6 mmol/L (129/16851)** | | | | | | |
| Event/Total | 10/4112 | 29/4143 | 44/4305 | 46/4291 |  |  |
| Incidence rate | 2.4 | 7.0 | 10.2 | 10.7 |  |  |
| Unadjusted model | Reference | 2.89 (1.41–5.92) | 4.22 (2.13–8.39) | 4.44 (2.24–8.80) | <0.001 | 1.60 (1.36–1.88) |
| Multivariable model | Reference | 2.53 (1.23–5.20) | 3.49 (1.74–6.97) | 3.54 (1.76–7.09) | <0.0001 | 1.50 (1.27–1.79) |
| **2.6≤LDL-C<3.4 mmol/L (209/17331)** | | | | | | |
| Event/Total | 30/4436 | 44/4341 | 58/4305 | 77/4249 |  |  |
| Incidence rate | 6.8 | 10.2 | 13.5 | 18.3 |  |  |
| Unadjusted model | Reference | 1.50 (0.94–2.39) | 2.00 (1.29–3.10) | 2.69 (1.77–4.11) | <0.001 | 1.39 (1.22–1.59) |
| Multivariable model | Reference | 1.27 (0.79–2.02) | 1.53 (0.98–2.40) | 1.97 (1.27–3.04) | <0.001 | 1.26 (1.09–1.46) |
| **LDL-C ≥3.4 mmol/L (137/6906)** | | | | | | |
| Event/Total | 28/1768 | 36/1844 | 45/1738 | 28/1556 |  |  |
| Incidence rate | 16.2 | 19.8 | 26.4 | 18.3 |  |  |
| Unadjusted model | Reference | 1.24 (0.75–2.02) | 1.64 (1.03–2.63) | 1.14 (0.67–1.92) | 0.351 | 1.06 (0.90–1.26) |
| Multivariable model | Reference | 1.10 (0.67–1.80) | 1.39 (0.86–2.26) | 0.94 (0.55–1.62) | 0.897 | 0.99 (0.82–1.19) |
| *P*-interaction: CumMHR quartiles*LDL-C (<1.8, 1.8 to 2.6, 2.6 to 3.4, or ≥3.4 mmol/L) = 0.057 | | | | | | |

The multivariable model was adjusted for sex, age (continuous), BMI (continuous), smoking habits (never, ever, current), alcohol consumption (yes or no), education level (less than high school, high school and above), physical activity (infrequent, occasional, frequent), family history of CVD (yes or no), loghsCRP (continuous), SBP (continuous), diabetes (yes or no), antihypertensives (yes or no), antidiabetics (yes or no), lipid-lowering drugs (yes or no).

Per SD: risk per SD increase in log (CumMHR) (0.194).

The incidence rate is per 1,0000 person-years.

Abbreviations: CumMHR, time-averaged cumulative monocyte-to-high density lipoprotein ratio; others were as in eTable 1.

# **eTable 7.** CumMHR-associated risk of incident MI according to sex (male/female)

|  | **CumMHR, HRs (95% CIs)** | | | | ***P*-trend** | **Per SD** |
| --- | --- | --- | --- | --- | --- | --- |
|  | **Quartile 1** | **Quartile 2** | **Quartile 3** | **Quartile 4** |  |  |
| **Male (520/37692)** | | | | | | |
| Event/Total | 66/7897 | 112/9211 | 160/10022 | 182/10562 |  |  |
| Incidence rate | 8.3 | 12.2 | 16.0 | 17.4 |  |  |
| Unadjusted model | Reference | 1.46 (1.08–1.98) | 1.92 (1.44–2.56) | 2.08 (1.57–2.76) | <0.001 | 1.29 (1.18–1.41) |
| Multivariable model | Reference | 1.41 (1.04–1.91) | 1.83 (1.37–2.45) | 1.97 (1.48–2.63) | <0.001 | 1.28 (1.17–1.40) |
| **Female (53/10830)** | | | | | | |
| Event/Total | 14/4233 | 15/2920 | 10/2108 | 14/1569 |  |  |
| Incidence rate | 3.3 | 5.2 | 4.8 | 9.0 |  |  |
| Unadjusted model | Reference | 1.54 (0.74–3.20) | 1.42 (0.63–3.19) | 2.67 (1.27–5.60) | 0.018 | 1.30 (1.01–1.67) |
| Multivariable model | Reference | 1.33 (0.64–2.79) | 1.19 (0.52–2.73) | 2.04 (0.94–4.43) | 0.112 | 1.18 (0.90–1.55) |
| *P*-interaction: CumMHR quartiles *sex (male or female) = 0.529 | | | | | | |

The multivariable model was adjusted for age (continuous), BMI (continuous), smoking habits (never, ever, current), alcohol consumption (yes or no), education level (less than high school, high school and above), physical activity (infrequent, occasional, frequent), family history of CVD (yes or no), TC (continuous), SBP (continuous), diabetes (yes or no), antihypertensives (yes or no), antidiabetics (yes or no), lipid-lowering drugs (yes or no), loghsCRP (continuous).

Per SD: risk per SD increase in log (CumMHR) (0.194).

The incidence rate is per 1,0000 person-years.

Abbreviations: CumMHR, time-averaged cumulative monocyte-to-high density lipoprotein ratio; others were as in eTable 1.

# **eTable 8.** CumMHR-associated risk of incident MI according to age subgroups (<55/≥55 years)

|  | **CumMHR, HRs (95% CIs)** | | | | ***P*-trend** | **Per SD** |
| --- | --- | --- | --- | --- | --- | --- |
|  | **Quartile 1** | **Quartile 2** | **Quartile 3** | **Quartile 4** |  |  |
| **Age<55 years (205/27354)** | | | | | | |
| Event/Total | 29/6932 | 45/6686 | 62/6696 | 69/7040 |  |  |
| Incidence rate | 4.1 | 6.7 | 9.2 | 9.7 |  |  |
| Unadjusted model | Reference | 1.61 (1.01–2.57) | 2.22 (1.43–3.45) | 2.36 (1.53–3.64) | <0.001 | 1.29 (1.13–1.48) |
| Multivariable model | Reference | 1.51 (0.95–2.42) | 1.93 (1.24–3.02) | 1.81 (1.16–2.83) | 0.008 | 1.17 (1.01–1.34) |
| **Age≥55 years (368/21168)** | | | | | | |
| Event/Total | 51/5198 | 82/5445 | 108/5434 | 127/5091 |  |  |
| Incidence rate | 10.0 | 15.3 | 20.2 | 25.5 |  |  |
| Unadjusted model | Reference | 1.54 (1.08–2.18) | 2.03 (1.46–2.83) | 2.56 (1.85–3.54) | <0.001 | 1.40 (1.27–1.55) |
| Multivariable model | Reference | 1.39 (0.98–1.97) | 1.74 (1.24–2.44) | 2.10 (1.50–2.94) | <0.001 | 1.33 (1.19–1.48) |
| *P*-interaction: time-averaged MHR quartile* age subgroups (<55 or ≥55 years) = 0.204 | | | | | | |

The multivariable adjusted model was adjusted for sex (male, female), BMI (continuous), smoking habits (never, ever, current), alcohol consumption (yes or no), education level (less than high school, high school and above), physical activity (infrequent, occasional, frequent), family history of CVD (yes or no), TC (continuous), SBP (continuous), loghsCRP (continuous), diabetes (yes or no), antihypertensives (yes or no), antidiabetics (yes or no), lipid-lowering drugs (yes or no).

Per SD: risk per SD increase in log (CumMHR) (0.194).

The incidence rate is per 1,0000 person-years.

Abbreviations: CumMHR, time-averaged cumulative monocyte-to-high density lipoprotein ratio; others were as in eTable 1.

# **eTable 9.** CumMHR-associated risk of incident MI according to prevalent hypertension (yes or no)

|  | **CumMHR, HRs (95% CIs)** | | | | ***P*-trend** | **Per SD** |
| --- | --- | --- | --- | --- | --- | --- |
|  | **Quartile 1** | **Quartile 2** | **Quartile 3** | **Quartile 4** |  |  |
| **Hypertension (473/30269)** | | | | | | |
| Event/Total | 69/6652 | 100/7409 | 136/7992 | 168/8216 |  |  |
| Incidence rate | 10.4 | 13.6 | 17.1 | 20.6 |  |  |
| Unadjusted model | Reference | 1.30 (0.96–1.77) | 1.65 (1.23–2.20) | 1.98 (1.50–2.62) | <0.001 | 1.29 (1.18–1.41) |
| Multivariable model | Reference | 1.23 (0.91–1.68) | 1.52 (1.14–2.05) | 1.80 (1.35–2.41) | <0.001 | 1.27 (1.14–1.38) |
| **Non-hypertension (100/18253)** | | | | | | |
| Event/Total | 11/5478 | 27/4722 | 34/4138 | 28/3915 |  |  |
| Incidence rate | 2.0 | 5.7 | 8.2 | 7.2 |  |  |
| Unadjusted model | Reference | 2.84 (1.41–5.73) | 4.08 (2.07–8.06) | 3.56 (1.78–7.16) | <0.001 | 1.39 (1.16–1.67) |
| Multivariable model | Reference | 2.43 (1.20–4.92) | 3.24 (1.63–6.44) | 2.78 (1.36–5.70) | 0.005 | 1.29 (1.05–1.57) |
| *P*-interaction: CumMHR quartiles*hypertension (yes or no) = 0.157 | | | | | | |

The multivariable model was adjusted for sex, age (continuous), BMI (continuous), smoking habits (never, ever, current), alcohol consumption (yes or no), education level (less than high school, high school and above), physical activity (infrequent, occasional, frequent), family history of CVD (yes or no), TC (continuous), loghsCRP (continuous), diabetes (yes or no), antihypertensives (yes or no), antidiabetics (yes or no), lipid-lowering drugs (yes or no).

Per SD: risk per SD increase in log (CumMHR) (0.194).

The incidence rate is per 1,0000 person-years.

Abbreviations: CumMHR, time-averaged cumulative monocyte-to-high density lipoprotein ratio; others were as in eTable 1.

# **eTable 10.** CumMHR-associated risk of incident MI according to prevalent diabetes (yes or no)

|  | **CumMHR, HRs (95% CIs)** | | | | ***P*-trend** | **Per SD** |
| --- | --- | --- | --- | --- | --- | --- |
|  | **Quartile 1** | **Quartile 2** | **Quartile 3** | **Quartile 4** |  |  |
| **Diabetes (148/7082)** | | | | | | |
| Event/Total | 18/1494 | 32/1721 | 44/1873 | 54/1994 |  |  |
| Incidence rate | 12.1 | 18.8 | 23.7 | 27.4 |  |  |
| Unadjusted model | Reference | 1.55 (0.87–2.76) | 1.96 (1.13–3.39) | 2.27 (1.33–3.86) | 0.002 | 1.33 (1.13–1.56) |
| Multivariable model | Reference | 1.52 (0.85–2.71) | 1.90 (1.09–3.31) | 2.18 (1.26–3.76) | 0.003 | 1.31 (1.11–1.55) |
| **Non-diabetes (425/41440)** | | | | | | |
| Event/Total | 62/10636 | 95/10410 | 126/10257 | 142/10137 |  |  |
| Incidence rate | 5.8 | 9.1 | 12.3 | 14.1 |  |  |
| Unadjusted model | Reference | 1.57 (1.14–2.16) | 2.11 (1.56–2.86) | 2.41 (1.79–3.25) | <0.001 | 1.34 (1.22–1.47) |
| Multivariable model | Reference | 1.35 (0.98–1.87) | 1.72 (1.26–2.34) | 1.87 (1.38–2.55) | <0.001 | 1.25 (1.13–1.38) |
| *P*-interaction: CumMHR quartiles*diabetes (yes or no) = 0.995 | | | | | | |

The multivariable model was adjusted for sex, age (continuous), BMI (continuous), smoking habits (never, ever, current), alcohol consumption (yes or no), education level (less than high school, high school and above), physical activity (infrequent, occasional, frequent), family history of CVD (yes or no), TC (continuous), SBP (continuous), antihypertensives (yes or no), antidiabetics (yes or no), lipid-lowering drugs (yes or no).

Per SD: risk per SD increase in log (CumMHR) (0.194).

The incidence rate is per 1,0000 person-years.

Abbreviations: CumMHR, time-averaged cumulative monocyte-to-high density lipoprotein ratio; others were as in eTable 1.

# **eTable 11.** CumMHR-associated risk of incident MI according to prevalent dyslipidemia (yes or no)

|  | **CumMHR, HRs (95% CIs)** | | | | ***P*-trend** | **Per SD** |
| --- | --- | --- | --- | --- | --- | --- |
|  | **Quartile 1** | **Quartile 2** | **Quartile 3** | **Quartile 4** |  |  |
| **Dyslipidemia (235/13905)** | | | | | | |
| Event/Total | 37/2885 | 48/3216 | 68/3521 | 82/4283 |  |  |
| Incidence rate | 12.9 | 15.0 | 19.5 | 19.4 |  |  |
| Unadjusted model | Reference | 1.16 (0.76–1.78) | 1.51 (1.01–2.25) | 1.50 (1.02–2.21) | 0.021 | 1.17 (1.04–1.33) |
| Multivariable model | Reference | 1.05 (0.68–1.62) | 1.24 (0.83–1.87) | 1.17 (0.79–1.75) | 0.352 | 1.09 (0.95–1.24) |
| **Non-dyslipidemia (338/34617)** | | | | | | |
| Event/Total | 43/9245 | 79/8915 | 102/8609 | 114/7848 |  |  |
| Incidence rate | 4.7 | 8.9 | 11.9 | 14.6 |  |  |
| Unadjusted model | Reference | 1.91 (1.32–2.77) | 2.55 (1.79–3.65) | 3.14 (2.21–4.45) | <0.001 | 1.44 (1.29–1.60) |
| Multivariable model | Reference | 1.60 (1.10–2.33) | 1.99 (1.39–2.85) | 2.32 (1.62–3.32) | <0.001 | 1.31 (1.17–1.47) |
| *P*-interaction: CumMHR quartiles*dyslipidemia (yes or no) = 0.069 | | | | | | |

The multivariable model was adjusted for sex, age (continuous), BMI (continuous), smoking habits (never, ever, current), alcohol consumption (yes or no), education level (less than high school, high school and above), physical activity (infrequent, occasional, frequent), family history of CVD (yes or no), loghsCRP (continuous), SBP (continuous), diabetes (yes or no), antihypertensives (yes or no), antidiabetics (yes or no), lipid-lowering drugs (yes or no).

Per SD: risk per SD increase in log (CumMHR) (0.194).

The incidence rate is per 1,0000 person-years.

Abbreviations: CumMHR, time-averaged cumulative monocyte-to-high density lipoprotein ratio; others were as in eTable 1.

# **eTable 12.** CumMHR-associated risk of incident MI according to renal dysfunction (yes or no)

|  | **CumMHR, HRs (95% CIs)** | | | | ***P*-trend** | **Per SD** |
| --- | --- | --- | --- | --- | --- | --- |
|  | **Quartile 1** | **Quartile 2** | **Quartile 3** | **Quartile 4** |  |  |
| **Renal dysfunction (328/21923)** | | | | | | |
| Event/Total | 37/4023 | 68/5256 | 108/6173 | 115/6471 |  |  |
| Incidence rate | 9.3 | 13.0 | 17.6 | 17.9 |  |  |
| Unadjusted model | Reference | 1.41 (0.94–2.10) | 1.90 (1.31–2.76) | 1.93 (1.34–2.80) | 0.001 | 1.22 (1.09–1.37) |
| Multivariable model | Reference | 1.33 (0.89–1.99) | 1.78 (1.21–2.60) | 1.74 (1.19–2.55) | 0.002 | 1.18 (1.05–1.33) |
| **Normal renal function (245/26599)** | | | | | | |
| Event/Total | 43/8107 | 59/6875 | 62/5957 | 81/5660 |  |  |
| Incidence rate | 5.3 | 8.6 | 10.4 | 14.4 |  |  |
| Unadjusted model | Reference | 1.62 (1.10–1.40) | 1.97 (1.34–2.91) | 2.73 (1.88–3.95) | <0.001 | 1.43 (1.27–1.61) |
| Multivariable model | Reference | 1.44 (0.97–2.14) | 1.64 (1.11–1.43) | 2.20 (1.50–3.23) | <0.001 | 1.35 (1.19–1.53) |
| *P*-interaction: CumMHR quartiles*renal dysfunction (yes or no) = 0.4947 | | | | | | |

The multivariable model was adjusted for sex, age (continuous), BMI (continuous), smoking habits (never, ever, current), alcohol consumption (yes or no), education level (less than high school, high school and above), physical activity (infrequent, occasional, frequent), family history of CVD (yes or no), TC (continuous), SBP (continuous), diabetes (yes or no), antihypertensives (yes or no), antidiabetics (yes or no), lipid-lowering drugs (yes or no), log(hsCRP) (continuous).

Per SD: risk per SD increase in log (CumMHR) (0.194).

The incidence rate is per 1,0000 person-years.

Abbreviations: CumMHR, time-averaged cumulative monocyte-to-high density lipoprotein ratio; others were as in eTable 1.

# **eTable 13.** CumMHR-associated risk of incident MI according to subclinical inflammation (hsCRP<2/≥2mg/L)

|  | **CumMHR, HRs (95% CIs)** | | | | ***P*-trend** | **Per SD** |
| --- | --- | --- | --- | --- | --- | --- |
|  | **Quartile 1** | **Quartile 2** | **Quartile 3** | **Quartile 4** |  |  |
| **Baseline hsCRP≥2 mg/L (249/15431)** | | | | | | |
| Event/Total | 25/2880 | 54/3715 | 79/4176 | 91/4660 |  |  |
| Incidence rate | 8.7 | 14.6 | 19.0 | 19.6 |  |  |
| Unadjusted model | Reference | 1.68 (1.04–2.69) | 2.19 (1.39–2.43) | 2.26 (1.45–3.52) | <0.001 | 1.33 (1.17–1.51) |
| Multivariable model | Reference | 1.55 (0.96–2.50) | 1.90 (1.21–3.00) | 2.49 (1.22–2.83) | 0.003 | 1.30 (1.13–1.48) |
| **Baseline hsCRP<2 mg/L (324/33091)** | | | | | | |
| Event/Total | 55/9250 | 73/8416 | 91/7954 | 105/7471 |  |  |
| Incidence rate | 6.0 | 8.7 | 11.5 | 14.2 |  |  |
| Unadjusted model | Reference | 1.46 (1.03–2.27) | 1.93 (1.38–2.70) | 2.38 (1.71–3.29) | <0.001 | 1.32 (1.19–1.47) |
| Multivariable model | Reference | 1.29 (0.91–1.83) | 1.65 (1.17–2.32) | 1.93 (1.38–2.70) | <0.001 | 1.24 (1.10–1.38) |
| *P*-interaction: CumMHR quartiles*baseline CRP (<2 or ≥2 mg/L) = 0.837 | | | | | | |

The multivariable model was adjusted for sex, age (continuous), BMI (continuous), smoking habits (never, ever, current), alcohol consumption (yes or no), education level (less than high school, high school and above), physical activity (infrequent, occasional, frequent), family history of CVD (yes or no), TC (continuous), SBP (continuous), diabetes (yes or no), antihypertensives (yes or no), antidiabetics (yes or no), lipid-lowering drugs (yes or no).

Per SD: risk per SD increase in log (CumMHR) (0.194).

The incidence rate is per 1,0000 person-years.

Abbreviations: CumMHR, time-averaged cumulative monocyte-to-high density lipoprotein ratio; others were as in eTable 1.

# **eTable 14.** Reverse analysis of CumMHR-associated risk of incident MI in the entire cohort (520/48469)

|  | **CumMHR, HRs (CIs)** | | | | **P-trend** | **Per SD** |
| --- | --- | --- | --- | --- | --- | --- |
|  | **Quartile 1** | **Quartile 2** | **Quartile 3** | **Quartile 4** |  |  |
| **Incident MI (520/48469)** | | | | | | |
| Event/Total | 76/12126 | 116/12120 | 154/12114 | 174/12109 |  |  |
| Incidence rate | 6.3 | 9.6 | 12.8 | 14.4 |  |  |
| Unadjusted model | Reference | 1.53 (1.14–2.04) | 2.03 (1.54–2.67) | 2.30 (1.76–3.01) | <0.001 | 1.34 (1.23–1.45) |
| Multivariable model | Reference | 1.35 (1.01–1.81) | 1.70 (1.28–2.24) | 1.85 (1.40–2.44) | <0.001 | 1.25 (1.14–1.37) |

The multivariable model was adjusted for sex, age (continuous), BMI (continuous), smoking habits (never, ever, current), alcohol consumption (yes or no), education level (less than high school, high school and above), physical activity (infrequent, occasional, frequent), family history of CVD (yes or no), TC (continuous), SBP (continuous), diabetes (yes or no), antihypertensives (yes or no), antidiabetics (yes or no), lipid-lowering drugs (yes or no).

The incidence rate is per 1,0000 person-years.

Per SD: risk per SD increase in log (CumMHR) (0.194).

Abbreviations: CumMHR, time-averaged cumulative monocyte-to-high density lipoprotein ratio; others were as in eTable 1.

# **eTable 15.** Fine-Gray model of CumMHR-associated risk of incident MI in the entire cohort

|  | **CumMHR, HRs (95% CIs)** | | | | ***P*-trend** | **Per SD** |
| --- | --- | --- | --- | --- | --- | --- |
|  | **Quartile 1** | **Quartile 2** | **Quartile 3** | **Quartile 4** |  |  |
| Unadjusted model | Reference | 1.59 (1.20–2.10) | 2.13 (1.63–2.78) | 2.46 (1.90–3.19) | <0.001 | 1.32 (1.23–1.42) |
| Multivariable model | Reference | 1.40 (1.06–1.86) | 1.77 (1.36–2.31) | 1.97 (1.51–2.57) | <0.001 | 1.27 (1.17–1.37) |

The multivariable model was adjusted for sex, age (continuous), BMI (continuous), smoking habits (never, ever, current), alcohol consumption (yes or no), education level (less than high school, high school and above), physical activity (infrequent, occasional, frequent), family history of CVD (yes or no), TC (continuous), SBP (continuous), diabetes (yes or no), antihypertensives (yes or no), antidiabetics (yes or no), lipid-lowering drugs (yes or no).

The incidence rate is per 1,0000 person-years.

Per SD: risk per SD increase in log (CumMHR) (0.194).

Abbreviations: CumMHR, time-averaged cumulative monocyte-to-high density lipoprotein ratio; others were as in eTable 1.

# **eTable 16.** CumMHR-associated risk of incident MI in the entire cohort on unimputed data (560/47693)

|  | **CumMHR, HRs (95% CIs)** | | | | ***P*-trend** | **Per SD** |
| --- | --- | --- | --- | --- | --- | --- |
|  | **Quartile 1** | **Quartile 2** | **Quartile 3** | **Quartile 4** |  |  |
| Unadjusted model | Reference | 1.59 (1.20–2.10) | 2.13 (1.63–2.78) | 2.46 (1.90–3.19) | <0.001 | 1.35 (1.25–1.46) |
| Multivariable model | Reference | 1.41 (1.06–1.88) | 1.75 (1.33–2.31) | 1.99 (1.52–1.61) | <0.001 | 1.27 (1.16–1.38) |

The multivariable model was adjusted for sex, age (continuous), BMI (continuous), smoking habits (never, ever, current), alcohol consumption (yes or no), education level (less than high school, high school and above), physical activity (infrequent, occasional, frequent), family history of CVD (yes or no), TC (continuous), SBP (continuous), diabetes (yes or no), antihypertensives (yes or no), antidiabetics (yes or no), lipid-lowering drugs (yes or no).

The incidence rate is per 1,0000 person-year.

Per SD: risk per SD increase in log (CumMHR) (0.194).

Abbreviations: CumMHR, time-averaged cumulative monocyte-to-high density lipoprotein ratio; others were as in eTable 1.

# **eTable 17.** Sensitivity analysis by adjusting for covariates only with SMD>0.1

|  | **Exposure to CumMON, CumHDL and CumMHR, HRs (95% CIs)** | | | | ***P*-trend** | **Per SD** |
| --- | --- | --- | --- | --- | --- | --- |
|  | Quartile 1 | Quartile 2 | Quartile 3 | Quartile 4 |  |  |
| **Exposure to CumMON** | | | | | | |
| Multivariable model | Reference | 1.59 (1.21–2.10) | 1.49 (1.13–1.98) | 1.54 (1.15–2.07) | 0.031 | 1.12 (1.02–1.23) |
| **Exposure to CumHDL** | | | | | | |
| Multivariable model | Reference | 0.82 (0.66–1.03) | 0.75 (0.60–0.94) | 0.67 (0.52–0.85) | <0.001 | 0.85 (0.78–0.93) |
| **Exposure to CumMHR** | | | | | | |
| Multivariable model | Ref. | 1.35 (1.02–1.80) | 1.66 (1.26–2.20) | 1.81 (1.36–2.42) | <0.001 | 1.24 (1.13–1.36) |

The multivariable model was adjusted for sex, age (continuous), BMI (continuous), smoking habits (never, ever, current), education level (less than high school, high school and above), physical activity (infrequent, occasional, frequent), SBP (continuous), TC (continuous), log (TG) (continuous), log(leucocyte count) (continuous), log(hsCRP) (continuous), diabetes (yes or no), antihypertensives (yes or no), antidiabetics (yes or no), lipid-lowering drugs (yes or no).


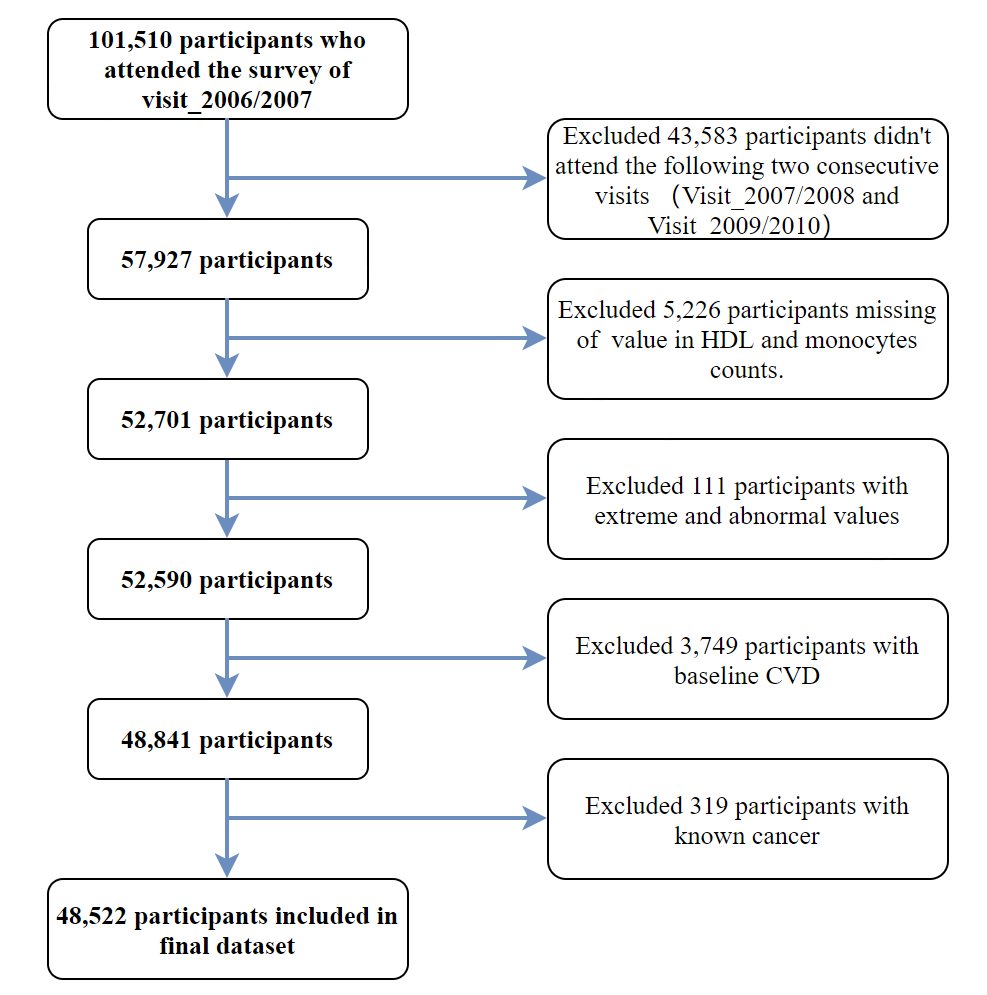


# **eFigure 1.** Flowchart of the study participants

Among 101,510 participants who attended the first health examination in 2006/2007, we excluded those did not attended the following two consecutive health surveys in 2007/2008 and 2009/2010 (n=43,583), those with incomplete data (n=5,226) or extreme values (n=111) in monocyte count and HDL-C levels in all three health examinations during exposure period, those with baseline CVD (n=3,749) or cancer (n=319), leaving a total of 48,522 participants in the analysis.


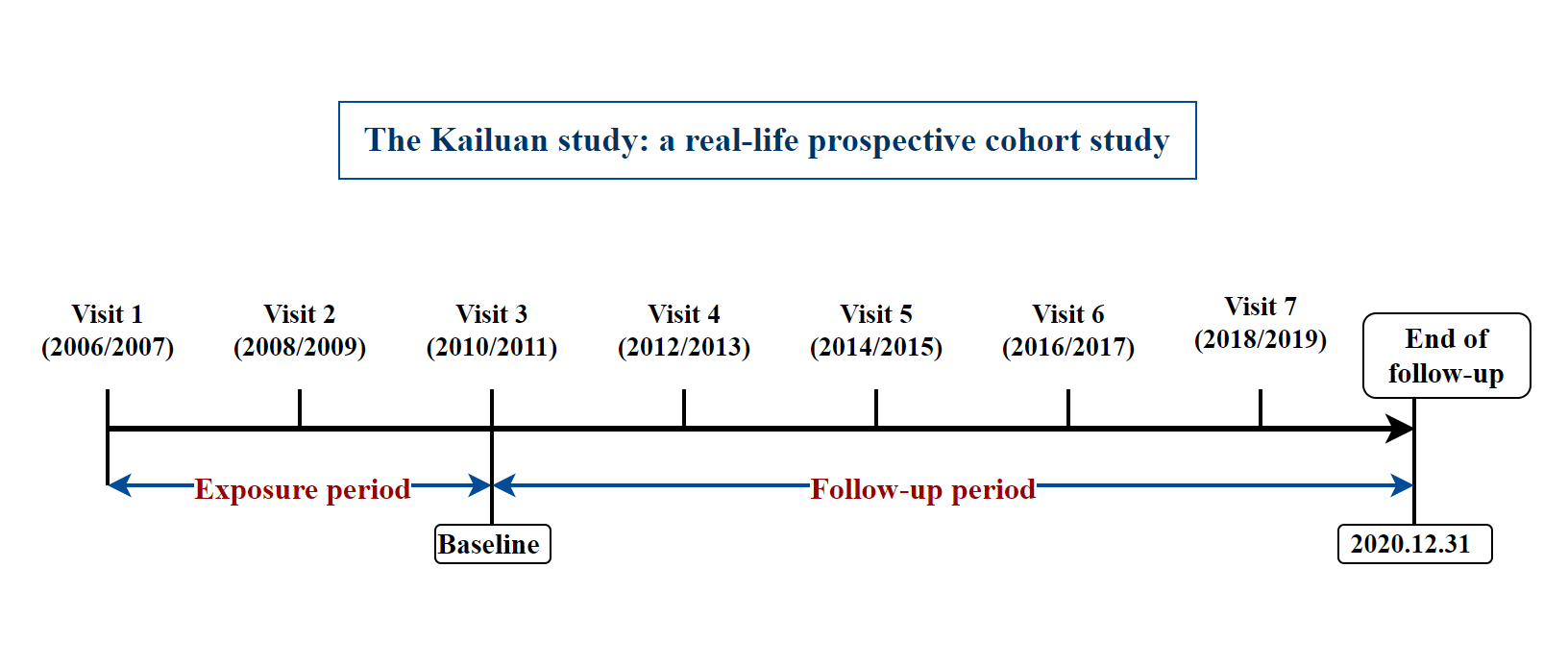


# **eFigure 2.** Study design of the present analysis

For the current analysis, time‑averaged cumulative exposure was calculated using data from three consecutive health examinations conducted between Visit 1 (2006/2007) and Visit 3 (2010/2011). Baseline was defined at Visit 3 (2010/2011), and follow‑up extended from 2010/2011 through December 31, 2020.


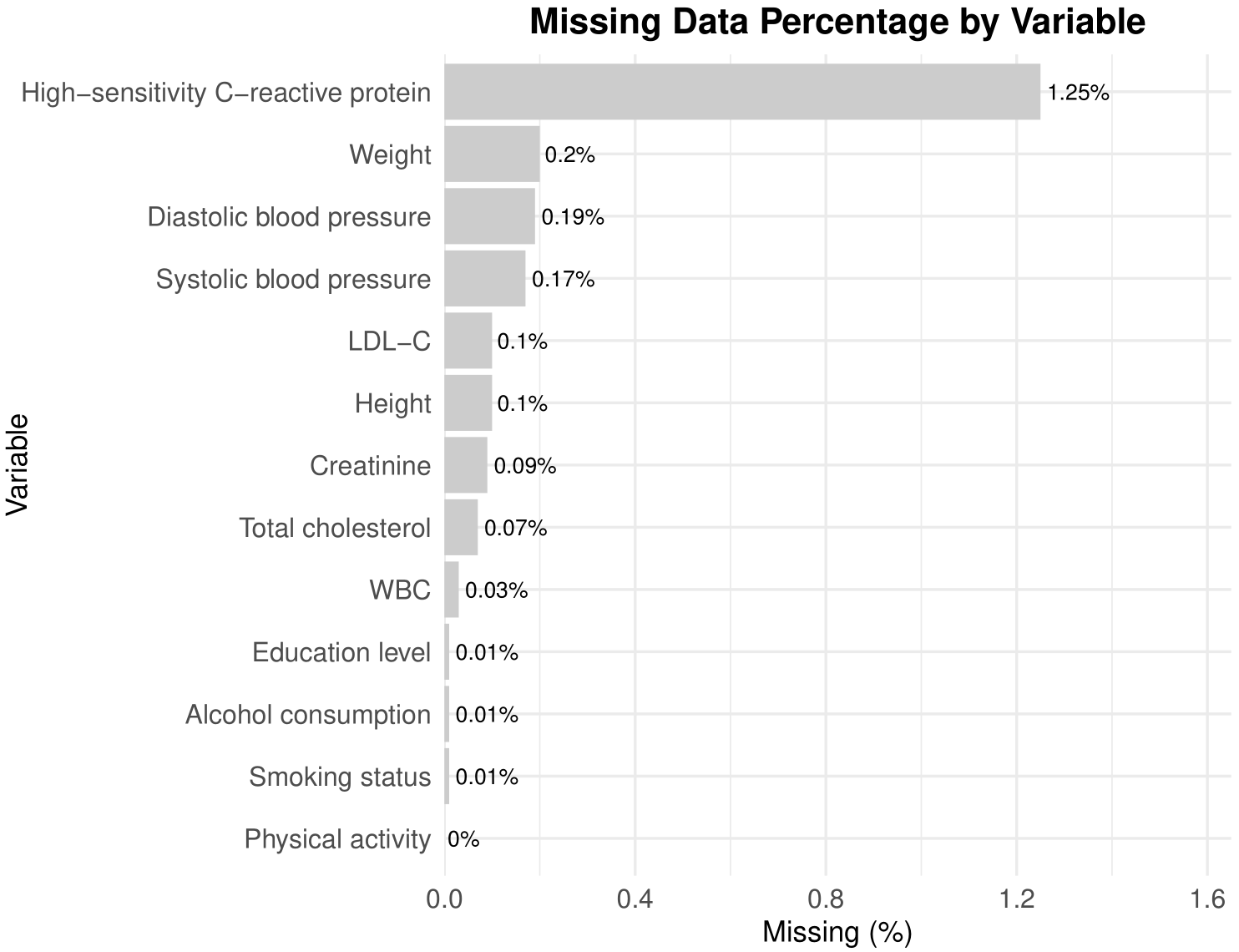


# **eFigure 3.** Missing rates of covariates


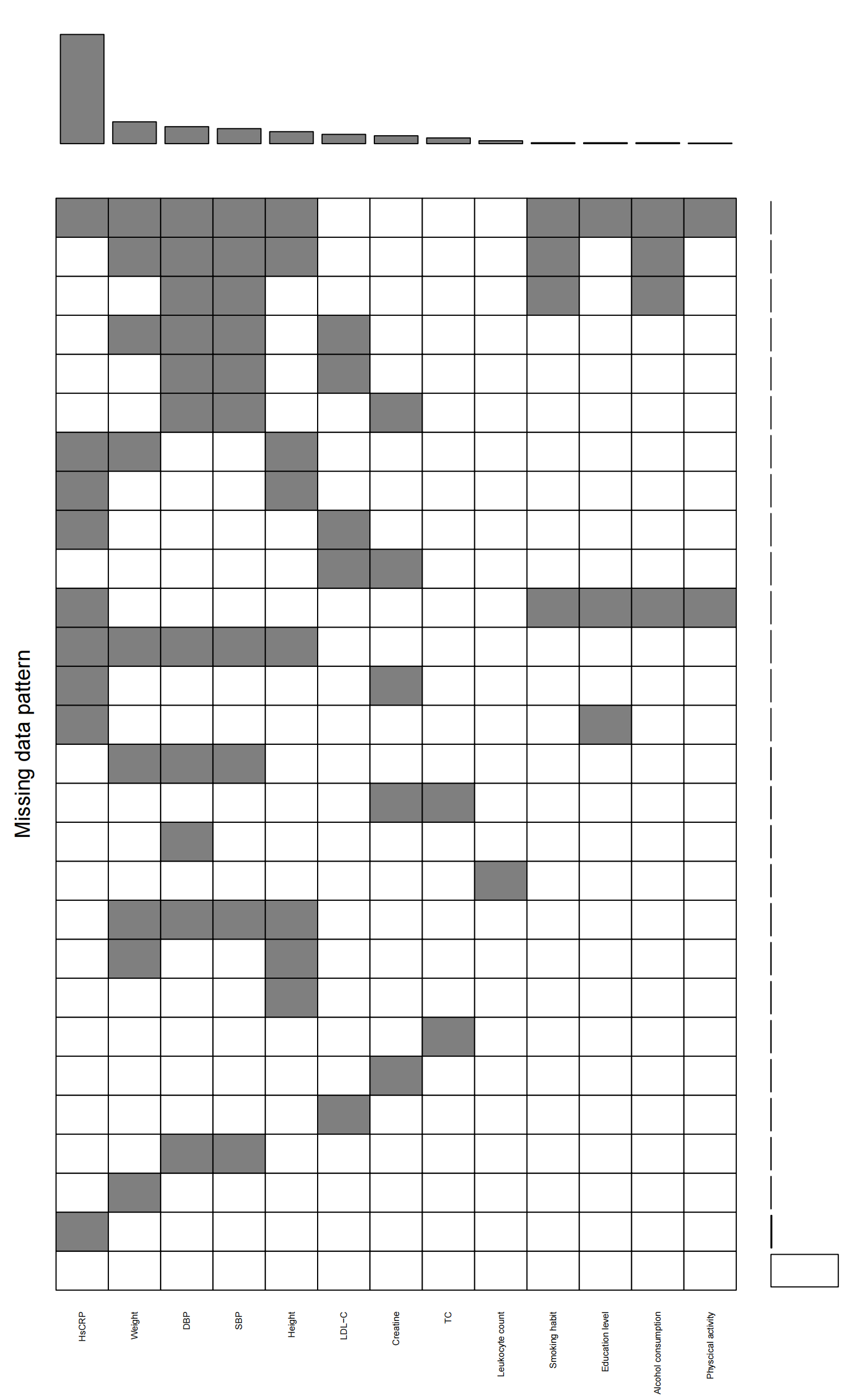


# **eFigure 4.** Missing pattern of covariates

# **Supplementary methods**

**Anthropometrics:**

Height and weight were measured by skilled staff using standardized procedures. Weight was assessed with participants wearing lightweight clothing and without shoes, and measurements were recorded to the nearest 0.1 kg. Participants' height was measured with a stadiometer, recorded to the nearest 0.1 cm. BMI was calculated as the weight in kilograms divided by the square of the height in meter. Blood pressure was measured from 7 to 9 am on the day of

physical examination. Smoking or drinking tea or coffee were prohibited within 30 minutes before the measurement. The systolic blood pressure (SBP) reading on desktop mercury sphygmomanometers was taken as the first-time phase of kirschmann sound, and the diastolic blood pressure (DBP) reading was taken as the fifth time phase of kirschmann sound. Measurement was repeated for 3 times, with each measurement interval of 1 to 2 minutes, and the mean value was taken.

**Lifestyle factors**:

Current drinker was defined as the consumption of ≥2 standard drinking volume/d for men and ≥1 standard drinking volume/d for women (1 standard drinking volume is equivalent to 120 ml of wine or 360 ml of beer or 45 ml of liquor) for 1 year and above [1]. Current smoker was defined as at least one cigarette per day in the past 1 year [1]. Physical activity were divided into three subgroups: infrequent (< 1 time/week), occational(1–2 times/ week), and frequent (≥ 3 times/week and ≥ 30 min/time) according to the self-reported the frequency of PA from questionnaire.[2]

**Laboratory tests:**

Fasting blood samples were collected in the morning after an 8 to 12 h overnight fasting and transfused into vacuum tubes containing EDTA. Biochemistry test on fasting blood glucose (FBG), lipid profiles, creatine, high-sensitivity C-reactive protein (hsCRP) was measured using automatic analyzer (Hitachi 747; Hitachi, Tokyo, Japan) at the central laboratory of Kailuan hospital, within 4 h after the collection of fasting blood samples.

**Definition of prevalent diseases:**

Hypertension was defined as systolic blood pressure ≥140 mmHg and/or diastolic blood pressure ≥90 mmHg or having a history of hypertension or using antihypertensive drugs. Diabetes was documented by a fasting blood glucose level ≥7.0 mmol/L and/or <7.0 mmol/L but with a confirmed history of diabetes or medication use hypoglycemic drugs. The estimated glomerular filtration rate (eGFR) was calculated in accordance with the Chronic Kidney Disease Epidemiology Collaboration (CKD-EPI) formula [3]. Renal dysfunction was defined as an eGFR <90 mL/(min·1.73 m^2^)[4]. Dyslipidemia includes a large range of lipid abnormalities and may involve a combination of increased total cholesterol (≥240 mg/dL [6.20 mmol/L]), LDL-C (>160 mg/dL [4.13 mmol/L]), and triglyceride levels (>200 mg/dL [2.25 mmol/L]) or decreased HDL-C (<40 mg/dL [1.03 mmol/ L])[5].

**Reference**

1. Huang, S., et al., *Longitudinal study of alcohol consumption and HDL concentrations: a community-based study.* Am J Clin Nutr, 2017. **105**(4): p. 905-912.

2. Tian, Q., et al., *Moderate physical activity may not decrease the risk of cardiovascular disease in persistently overweight and obesity adults.* J Transl Med, 2022. **20**(1): p. 45.

3. Levey, A.S., et al., *A new equation to estimate glomerular filtration rate.* Ann Intern Med, 2009. **150**(9): p. 604-12.

4. Smith, G.L., et al., *Renal impairment and outcomes in heart failure: systematic review and meta-analysis.* J Am Coll Cardiol, 2006. **47**(10): p. 1987-96.

5. Kopin, L. and C. Lowenstein, *Dyslipidemia.* Ann Intern Med, 2017. **167**(11): p. Itc81-itc96.
